# The Cardiac Genome Clinic: implementing genome sequencing in pediatric heart disease

**DOI:** 10.1038/s41436-020-0757-x

**Published:** 2020-02-10

**Authors:** Miriam S. Reuter, Rajiv R. Chaturvedi, Eriskay Liston, Roozbeh Manshaei, Ritu B. Aul, Sarah Bowdin, Iris Cohn, Meredith Curtis, Priya Dhir, Robin Z. Hayeems, S. Mohsen Hosseini, Reem Khan, Linh G. Ly, Christian R. Marshall, Luc Mertens, John B. A. Okello, Sergio L. Pereira, Akshaya Raajkumar, Mike Seed, Bhooma Thiruvahindrapuram, Stephen W. Scherer, Raymond H. Kim, Rebekah K. Jobling

**Affiliations:** 1 Ted Rogers Centre for Heart Research, Cardiac Genome Clinic, The Hospital for Sick Children, Toronto, ON Canada; 2 CGEn, The Hospital for Sick Children, Toronto, ON Canada; 3 The Centre for Applied Genomics, The Hospital for Sick Children, Toronto, ON Canada; 4 Program in Genetics and Genome Biology, The Hospital for Sick Children, Toronto, ON Canada; 5 Labatt Heart Centre, Division of Cardiology, The Hospital for Sick Children, Toronto, ON Canada; 6 Division of Clinical and Metabolic Genetics, The Hospital for Sick Children, Toronto, ON Canada; 7 Divisions of Clinical Pharmacology and Toxicology, The Hospital for Sick Children, Toronto, ON Canada; 8 Faculty of Medicine, University of Toronto, Toronto, ON Canada; 9 Program in Child Health Evaluative Sciences, The Hospital for Sick Children, Toronto, ON Canada; 10 Division of Neonatology, The Hospital for Sick Children, Toronto, ON Canada; 11 Department of Paediatrics, The Hospital for Sick Children, Toronto, ON Canada; 12 Department of Paediatrics, University of Toronto, Toronto, ON Canada; 13 Laboratory Medicine and Pathobiology, University of Toronto, Toronto, ON Canada; 14 Genome Diagnostics, Department of Paediatric Laboratory Medicine, The Hospital for Sick Children, Toronto, ON Canada; 15 Department of Molecular Genetics, University of Toronto, Toronto, ON Canada; 16 Fred A. Litwin Family Centre in Genetic Medicine, University Health Network, Department of Medicine, University of Toronto, Toronto, ON Canada

**Keywords:** genome sequencing, exome sequencing, congenital heart disease, gene discovery, ACMG guidelines

## Abstract

**Purpose:**

This study investigated the diagnostic utility of nontargeted genomic testing in patients with pediatric heart disease.

**Methods:**

We analyzed genome sequencing data of 111 families with cardiac lesions for rare, disease-associated variation.

**Results:**

In 14 families (12.6%), we identified causative variants: seven were de novo (*ANKRD11*, *KMT2D*, *NR2F2*, *POGZ*, *PTPN11*, *PURA*, *SALL1*) and six were inherited from parents with no or subclinical heart phenotypes (*FLT4*, *DNAH9*, *MYH11*, *NEXMIF*, *NIPBL*, *PTPN11*). Outcome of the testing was associated with the presence of extracardiac features (*p* = 0.02), but not a positive family history for cardiac lesions (*p* = 0.67). We also report novel plausible gene–disease associations for tetralogy of Fallot/pulmonary stenosis (*CDC42BPA*, *FGD5*), hypoplastic left or right heart (*SMARCC1*, *TLN2*, *TRPM4*, *VASP*), congenitally corrected transposition of the great arteries (*UBXN10*), and early-onset cardiomyopathy (*TPCN1*). The identified candidate genes have critical functions in heart development, such as angiogenesis, mechanotransduction, regulation of heart size, chromatin remodeling, or ciliogenesis.

**Conclusion:**

This data set demonstrates the diagnostic and scientific value of genome sequencing in pediatric heart disease, anticipating its role as a first-tier diagnostic test. The genetic heterogeneity will necessitate large-scale genomic initiatives for delineating novel gene–disease associations.

## INTRODUCTION

Congenital heart disease (CHD) describes a heterogeneous set of disorders that affect the structure or function of the developing heart. With a birth prevalence of 1–3%, CHD is the most common congenital anomaly in humans.^1^ Childhood cardiomyopathies are progressive disorders and a common cause of heart failure in children.^2^ The causes of pediatric heart disease are diverse and often multifactorial. Evidence for major genetic contributions come from familial recurrence rates, twin studies, and a higher incidence in consanguineous populations.^3,4^ Mendelian forms of idiopathic CHD are considered rare, and many of the known loci are associated with incompletely penetrant, variable cardiac, and extracardiac manifestations. Clinical genetic assessments are not systematically offered to families with cardiac lesions, and there are no formal diagnostic testing protocols. Limited accession of genetic services and hypothesis-driven approaches may result in etiological underdiagnoses and/or diagnostic odysseys.

High-throughput (exome or genome) sequencing studies of cohorts with CHD had reported remarkably disparate diagnostic rates (5.2–43.3%), which also correlated with the stringency in variant interpretation.^5–8^ Compared with less comprehensive techniques, genome sequencing allows for an unbiased analysis of most types of genomic variation, and had a higher yield than standard of care genetic testing in clinically heterogeneous cohorts.^9^ However, variant interpretation may be challenging, particularly for sporadic disease with limited genotype–phenotype correlations and incomplete penetrance.

The Cardiac Genome Clinic was established to investigate the utility of genome sequencing in families with pediatric heart disease. As part of a pilot study, we obtained genome sequencing data of 111 unrelated probands (*n* = 107 sequenced as parent–child trios/quartets/extended families), and systematically analyzed for rare, predicted damaging variation (single-nucleotide variants, insertions/deletions and structural variants). Variants in disease-associated genes were interpreted according to standard guidelines,^10^ and novel candidate genes were prioritized according to their biological plausibility.

## MATERIALS AND METHODS

### Study participants

**Fig. 1:**
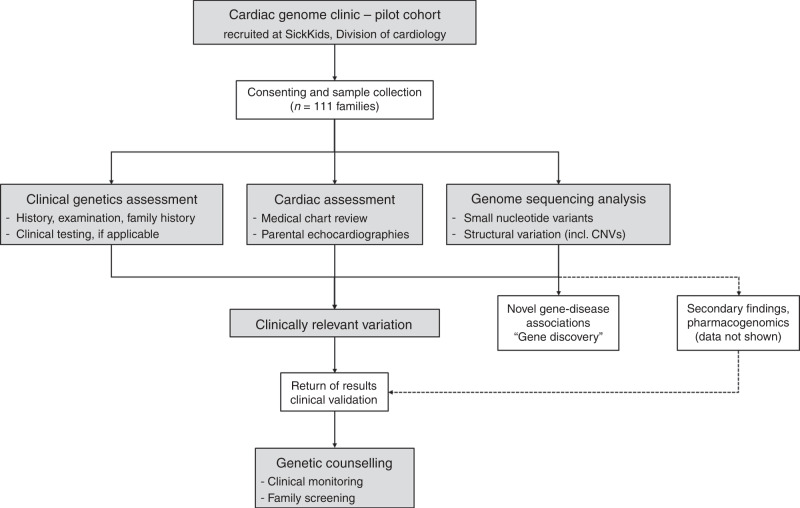
Concept and process of the Cardiac Genome Clinic. Families with pediatric heart disease (*n* = 111) were recruited through the Division of Cardiology at The Hospital for Sick Children. The genome sequencing data was analyzed for small nucleotide and structural variation. Clinically relevant variants were returned to participants per consent. *CNV* copy-number variant.

The study was approved by the Research Ethics Board at The Hospital for Sick Children (REB #1000053844). Informed consent was obtained from all probands and family members. Study participants originated from a cohort of families with pediatric heart disease, recruited through the Ted Rogers Cardiac Genome Clinic at a single site, The Hospital for Sick Children, Division of Cardiology (from January 2017 to December 2018; Fig. 1, S1). By study design, families with laterality defects, outflow tract obstructions, or cardiomyopathies were preferentially enrolled. Exclusion criteria were known syndromes, metabolic diseases, or medical conditions leading to secondary heart failure. Phenotype data were entered into PhenoTips (https://phenotips.org/), using the Human Phenotype Ontology (https://hpo.jax.org/). If possible, we sequenced genomes of parent–child trios/quartets (*n* = 103), or multiple affected relatives (*n* = 4), resulting in a total of 328 sequenced individuals from 111 families.

### Genome sequencing and annotation

DNA was sequenced on the Illumina HiSeq X system at The Centre for Applied Genomics (TCAG) in Toronto, Canada (details on sequencing and data analysis as supplementary information). Genome sequencing was performed under a research protocol, not as a validated clinical test. Population allele frequencies were derived from 1000 Genomes (https://www.internationalgenome.org/), ExAC (http://exac.broadinstitute.org/), and gnomAD (https://gnomad.broadinstitute.org/). Gene constraint metrics were derived from ExAC (probability of loss-of-function intolerance; pLI) and gnomAD (pLI, observed over expected loss-of-function variants; o/e). Variant information was queried from PubMed (https://www.ncbi.nlm.nih.gov/pubmed/), DECIPHER (https://decipher.sanger.ac.uk/), the Human Gene Mutation Database (http://www.hgmd.cf.ac.uk/ac/index.php), and ClinVar (https://www.ncbi.nlm.nih.gov/clinvar/).

### Variant prioritization and interpretation

**Fig. 2:**
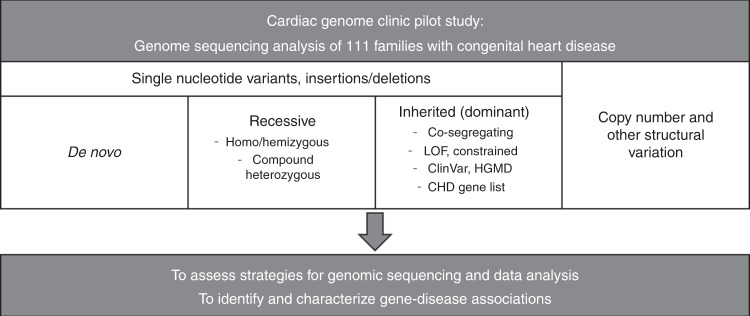
Systematic analysis of genome sequencing data. Different inheritance patterns were considered to allow a comprehensive assessment of genomic variation. *CHD* congenital heart disease, *HGMD* Human Gene Mutation Database, *LOF* loss of function.

We analyzed the data for various types of genomic variation (small variants affecting single genes, copy number, and other structural aberrations) and Mendelian inheritance patterns (de novo, recessive, and dominantly inherited, also considering incomplete penetrance; Fig. 2). Inherited variants were prioritized according to (1) cosegregation with disease, (2) previous reports in cardiovascular disease, (3) predicted loss-of-function of constrained genes (ExAC pLI ≥0.9), and (4) predicted damaging effects in CHD genes (http://chdgene.victorchang.edu.au/).^8^ Variants in genes known to be associated with cardiac disease were interpreted in accordance with clinical standards and guidelines of the American College of Medical Genetics and Genomics (ACMG).^10^ Likely pathogenic, pathogenic, and uncertain variants were reviewed by a clinical geneticist, a genetic counselor, and a cardiologist in the context of the phenotype and family history. For the diagnostic yield, we considered variants deemed “causative” for the CHD by the clinical assessment. For novel candidate genes, we assessed the biological and experimental plausibility based on a literature review. Copy-number variants (CNVs) were interpreted regarding a known or potential role in cardiovascular disorders. Variants of interest were confirmed through Sanger sequencing or clinical microarrays. Relevant findings were reported back to the families through a clinical geneticist and a genetic counselor, and were sent for clinical validation.

## RESULTS

### Cohort characteristics

We prospectively recruited 111 families with congenital heart disease or childhood-onset cardiomyopathies. Of those, 53 probands (47.7%) had extracardiac features, defined as other major malformations, intellectual disability, autism, global developmental delay, or growth deficits not attributable to heart failure. Thirteen families (11.7%) reported relatives with clinically relevant cardiac lesions, two probands (1.8%) had a parent with bicuspid aortic valve, and four parents (3.6%) were consanguineous (Table 1, S1). Ninety families (81.1%) were formally assessed by a clinical geneticist, 85 had parental echocardiography (*n* = 78 biparental; 70.3%), and 71 (64.0%) had negative standard of care genetic testing (such as chromosomal microarrays, targeted gene/panel testing, clinical exome sequencing; Table S1). The spectrum of primary cardiac phenotypes is displayed in Table 1, phenotypic details in Table S1.

**Table 1:** Characteristics of 111 index patients.

Category	*N*
All	111
Male sex	68
Primary cardiac lesion	
Aortic stenosis or arch obstruction (non-HLHS)	25
Tetralogy of Fallot	16
HLHS	15
Transposition of the great arteries	13
Univentricular heart (non-HLHS, nonlaterality defect)	10
Pulmonary stenosis/atresia^a^	8
Septal defect^b^	8
Laterality defect^c^	6
Cardiomyopathy	3
Other^d^	7
Cardiac family history	
Familial heart defect	13
Bicuspid aortic valve	2
Extracardiac features	53

Twenty-seven probands with univentricular heart: HLHS (15), laterality defect (2), tricuspid atresia (6), double inlet left ventricle (3), Ebstein anomaly (1).

*HLHS* hypoplastic left heart syndrome.

^a^Valvar pulmonary stenosis (5), valvar pulmonary atresia with intact ventricular septum (3).

^b^Atrioventricular septal defect (4), ventricular septal defect (2), atrial septal defect (2).

^c^Dextrocardia (5), right atrial isomerism (2), left atrial isomerism (2).

^d^Patent ductus arteriosus (2), isolated total anomalous pulmonary venous drainage (2), congenitally corrected transposition of the great arteries (2), common arterial trunk (1).

### Clinically relevant variants

To assess the diagnostic utility of genome sequencing in children with cardiac disease, we interpreted the data for clinically relevant variants. We identified causative variants in 14 of 111 families (12.6%); Table 2. Ten of the affected genes were found on a curated list of 107 high-confidence CHD-associated genes (http://chdgene.victorchang.edu.au/; December 2019). Eleven diagnoses were made in patients with extracardiac features (11/53 vs. 3/58; Fisher’s exact test (FET): *p* = 0.02), and two in patients with familial heart defects (2/13 vs. 12/98; FET: *p* = 0.67).

**Table 2:** Variants of potential clinical relevance.

ID	Cardiac lesion	Other anomalies	Gene(s)	Variant	Inheritance	Variant interpretation	Clinical assessment	Diagnosis
042	AVSD	Feeding difficulties, polyhydramnios, deceased at 7 months	*ANKRD11* (NM_013275.5)	c.5238_5239delGC, p.(Pro1747Argfs*49)	De novo	Pathogenic	Causative (CHD, other anomalies)^b^	KBG syndrome
087	Dextrocardia, left atrial isomerism, AVSD, hypoplastic LV, DORV	Macrocephaly, left-sided liver, polysplenia, right-sided stomach, ultrastructural ciliary abnormalities	*DNAH9* (NM_001372.3)	c.[4421A>G];[13244T>C], p.[(Asp1474Gly)];[(Ile4415Thr)]	Paternal; maternal	Uncertain significance	Causative (CHD, other anomalies)^b^	Primary ciliary dyskinesia
034	TOF, PA, MAPCAS	- (Mild congenital lymphedema)	*FLT4*^a^ (NM_182925.4)	c.89delC, p.(Pro30Argfs*3)	Maternal	Likely pathogenic	Causative (CHD)^b^	Nonsyndromic TOF
148	AS, CoA, VSD	Developmental delay, failure to thrive, dysmorphisms	*KMT2D* (NM_003482.3)	c.15673C>T, p.(Arg5225Cys)	De novo	Likely pathogenic	Causative (CHD, other anomalies)^b^	Kabuki syndrome
054	PDA	-	*MYH11* (NM_002474.2)	c.4578+1G>A, p.?	Paternal	Pathogenic	Causative (CHD)^b^	Familial PDA
027	AS	Developmental delay, microcephaly, short stature, hypotonia	*NEXMIF* (NM_001008537.2)	c.1502delG, p.(Gly501Valfs*4), hemizygous	Maternal (X-linked)	Likely pathogenic	Causative (CHD, other anomalies)^b^	NEXMIF-related developmental disorder
032	AVSD, hypoplastic right ventricle	Developmental delay, borderline microcephaly, hypotonia	*NIPBL* (NM_133433.3)	c.771+1G>A, p.?	Maternal	Pathogenic	Causative (CHD, other anomalies)^b^	Cornelia de Lange syndrome
033	TOF, PA	-	*NOTCH1* deletion (i)	9q34.3 deletion (138 kb)	Unknown	Pathogenic	Causative (CHD)^b^	NOTCH1-related CHD
039	CoA, BAV, muscular VSD	Intellectual disability, macrocephaly, hemangioma, scoliosis	*NR2F2* (NM_021005.3)	c.671T>A, p.(Val224Asp)	De novo	Likely pathogenic	Causative (CHD), possibly causative (other anomalies)^b^	NR2F2-related CHD
157	HLHS (mitral atresia, aortic atresia)	Developmental delay, borderline short stature, hypotonia	*POGZ* (NM_015100.3)	c.3403delG, p.(Glu1135Argfs*3)	De novo	Pathogenic	Causative (CHD, other anomalies)^b^	White–Sutton syndrome
018	PS	Borderline short stature	*PTPN11* (NM_002834.4)	c.209A>G, p.(Lys70Arg)	Maternal	Likely pathogenic	Causative (CHD, other anomalies)^b^	Noonan syndrome
060	CoA, VSD	Increased nuchal translucency, failure to thrive, short stature, dysmorphisms, hypotonia	*PTPN11* (NM_002834.4)	c.923A>G, p.(Asn308Ser)	De novo	Pathogenic	Causative (CHD, other anomalies)^b^	Noonan syndrome
013	VSD	Developmental delay, infantile spasms, hypoventilation, macrosomia, macrocephaly, hypotonia	*PTEN* (NM_000314.4)	c.395G>A, p.(Gly132Asp)	De novo	Pathogenic	Causative (other anomalies)	PTEN hamartoma syndrome
			*PURA* (NM_005859.4)	c.812_814delTCT, p.(Phe271del)	De novo	Pathogenic	Causative (CHD, other anomalies)^b^	PURA-related developmental disorder
074	Interrupted aortic arch type B, large VSD	Borderline short stature, hearing impairment, dysplastic ears with preauricular tags	*SALL1* deletion (ii)	16q12.1-16q12.2 (4.1 Mb)	De novo	Pathogenic	Causative (CHD, other anomalies)^b^	Townes–Brocks syndrome
010	Hypertrophic cardiomyopathy		*FLNC* (NM_001458.4)	c.6238G>C, p.(Gly2080Arg)	Paternal	Uncertain significance	Likely causative	Hypertrophic cardiomyopathy
093	Dextrocardia, right atrial isomerism, AVSD, PA	Failure to thrive, short stature, borderline microcephaly, asplenia, right-sided stomach	*DNAH8* (NM_001206927.1)	c.[991A>G];[10773C>G], p.[(Thr331Ala)];[(Phe3591Leu)]	Paternal; maternal	Uncertain significance	Likely causative	Primary ciliary dyskinesia
			(iii)	3p11.2-3p12.3 deletion (8.3 Mb)	Maternal	Uncertain significance	Uncertain	Unknown
019	Interrupted aortic arch type B, AS	Apnea, microcephaly, dysmorphisms, anemia, hyponatremia, deceased at 2 months	(iv)	15q13.2-15q13.3 duplication (2.4 Mb)	Paternal	Uncertain significance	Uncertain	Unknown
024	Transposition of the great arteries	Mild intellectual disability, autism	(v)	16p13.3 duplication (697 kb)	Maternal	Uncertain significance	Uncertain	Unknown
099	Tricuspid valve dysplasia, hypoplastic right heart, VSD, ASD	Mild intellectual disability, ADHD, periauricular skin tag	(vi)	3p26.1-3pter deletion (4.3 Mb), 3p26.1 duplication (1.8 Mb)	Maternal	Uncertain significance	Uncertain	Unknown
149	CoA, BAV		(vii)	2p13.1-2p12 deletion (594 kb)	Paternal	Uncertain significance	Uncertain	Unknown
044	CoA, multiple VSD	Mild developmental delay, borderline macrocephaly	*PTEN* (NM_000314.6)	c.45A>C, p.(Arg15Ser)	Paternal	Pathogenic	Causative (other anomalies)	PTEN hamartoma syndrome
059	Large ASD secundum		*DSG2* deletion (viii)	18q12.1 (9.1 kb)	De novo	Likely pathogenic	Secondary finding	Arrhythmogenic right ventricular cardiomyopathy

Variants are heterozygous/compound heterozygous, unless indicated otherwise.

*ADHD* attention deficit–hyperactivity disorder, *AS* aortic stenosis, *ASD* atrial septal defect, *AVSD* atrioventricular septal defect, *BAV* bicuspid aortic valve, *CHD* congenital heart disease, *CoA* aortic coarctation, *DORV* double outlet right ventricle, *HLHS* hypoplastic left heart syndrome, *MAPCAS* major aortopulmonary collateral arteries, *PA* pulmonary atresia, *PDA* patent ductus arteriosus, *PS* pulmonic stenosis, *TOF* tetralogy of Fallot, *VSD* ventricular septal defect.

^a^Previously published Reuter et al.^15^

^b^Variants were considered for diagnostic yield.

(i) chr9:139345895–139484281 deletion of genes: *NOTCH1*, *SEC16A*, *C9orf163*.

(ii) chr16:49326510–53476612 deletion of genes: *SALL1*, *C16orf78*, *ZNF423*, *CNEP1R1*, *HEATR3*, *TENT4B*, *ADCY7*, *BRD7*, *NKD1*, *SNX20*, *NOD2*, *CYLD*, *C16orf97*, *TOX3*, *CHD9*, *LOC643802*, *RBL2*.

(iii) chr3:79166228–87437174 deletion of genes: *CADM2*, *CHMP2B*, *GBE1*, *POU1F1*, *ROBO1*, *VGLL3*.

(iv) chr15:30388001–32766000 duplication of genes: *GOLGA8T*, *CHRFAM7A*, *GOLGA8R*, *LOC100996413*, *GOLGA8Q*, *GOLGA8H*, *ARHGAP11B*, *FAN1*, *MTMR10*, *TRPM1*, *LOC283710*, *KLF13*, *OTUD7A*, *CHRNA7*, *GOLGA8K*, *GOLGA8O*.

(v) chr16:1828001–2525000 duplication of genes: *ABCA3*, *BRICD5*, *C16orf59*, *CASKIN1*, *CCNF*, *DNASE1L2*, *E4F1*, *ECI1*, *FAHD1*, *GFER*, *HAGH*, *HS3ST6*, *IGFALS*, *MEIOB*, *MLST8*, *MSRB1*, *NDUFB10*, *NOXO1*, *NPW*, *NTHL1*, *NTN3*, *NUBP2*, *PGP*, *PKD1*, *RAB26*, *RNF151*, *RNPS1*, *RPL3L*, *RPS2*, *SLC9A3R2*, *SPSB3*, *SYNGR3*, *TBL3*, *TRAF7*, *TSC2*, *ZNF598*.

(vi) chr3:60001–4354200 deletion of genes: *CHL1*, *CNTN4*, *CNTN6*, *CRBN*, *IL5RA*, *LRRN1*, *SETMAR*, *TRNT1*. chr3:4360001–6187000 duplication of genes: *ARL8B*, *BHLHE40*, *EDEM1*, *ITPR1*, *SUMF1*.

(vii) chr2:74646148–75240197 deletion of genes: *RTKN*, *WDR54*, *CCDC142*, *LBX2*, *DOK1*, *HTRA2*, *INO80B*, *AUP1*, *MOGS*, *WBP1*, *C2orf81*, *SEMA4F*, *M1AP*, *TTC31*, *LOXL3*, *INO80B-WBP1*, *TLX2*, *DQX1*, *POLE4*, *MRPL53*, *PCGF1*, *HK2*.

(viii) chr18:29073480–29082599 deletion of *DSG2* exon 1 and promoter region.

#### Small nucleotide variants

Seven individuals with prominent extracardiac anomalies and developmental delay had disease-causing de novo variants, such as p.(Pro1747Argfs*49) in *ANKRD11* (KBG syndrome), or p.(Arg5225Cys) in *KMT2D* (Kabuki syndrome). Both genes are associated with highly penetrant congenital heart defects. In a patient with ventricular septal defect (VSD), aortic coarctation, and neurological symptoms, we identified a de novo missense substitution p.(Val224Asp) in the ligand binding domain of NR2F2. Missense variants in *NR2F2* were associated with CHD (particularly septal defects) and a broad spectrum of associated anomalies.^11^ A de novo variant p.(Glu1135Argfs*3) in *POGZ* was found in a proband with hypoplastic left heart syndrome (HLHS) and developmental delay, supporting the gene’s pleiotropic effects in brain and heart development.^12,13^ A proband with VSD, developmental delay, hypotonia, respiratory issues, and growth anomalies had two de novo, recurrent variants p.(Phe271del) in *PURA* and p.(Gly132Asp) in *PTEN*. Both defects contribute to the phenotype, and a minority of patients with *PURA*-related disorders present with structural heart defects.^14^

In other cases of apparently sporadic CHD, pathogenic nucleotide variants were inherited from parents with no or subclinical heart disease (*n* = 5). A frameshift deletion p.(Pro30Argfs*3) in *FLT4* was identified in a patient with tetralogy of Fallot (TOF) and her unaffected mother.^15^ *FLT4* haploinsufficiency was recently associated with incompletely penetrant nonsyndromic TOF.^15–17^ A patient with aortic stenosis, valve dysplasia, and developmental delay had a variant p.(Gly501Valfs*4) in *NEXMIF*, which was X-linked inherited from the mother with mild intellectual disability and epilepsy. Cardiac defects are not common for *NEXMIF*-related disease, but valve dysfunctions (pulmonary stenosis, mitral insufficiency) were infrequently reported.^18^ In a patient with hypoplastic right heart, unbalanced septal defect, developmental delay, and borderline microcephaly, we identified a pathogenic *NIPBL* variant c.771+1G>A for Cornelia de Lange syndrome. The variant was inherited from the mother, who had short stature, small hands, and delayed menarche, but normal cognitive and cardiac presentation, indicating variable expressivity. The broad spectrum of cardiac lesions associated with *NIPBL* haploinsufficiency likely results from subtle transcriptional dysregulations of hundreds of genes.^19^ A patient with dysplastic aortic and pulmonary valve and borderline short stature was identified with a maternally inherited *PTPN11* variant p.(Lys70Arg) for Noonan syndrome. The mother was considered healthy; however, a research echo at the time of study enrollment showed decreased ventricular function of unknown origin. A paternally inherited, pathogenic *MYH11* variant c.4578+1G>A, resulting in an in-frame loss of 71 amino acids,^20^ was identified in a proband with patent ductus arteriosus (PDA). Cosegregation testing in four paternal relatives with PDA and a grandfather with aortic disease in his eighties could not be performed. The same protein change was reported in an unrelated family with familial PDA.^21^ Further phenotype–genotype correlations are required to provide risk estimates for aortic disease in such families. In a female patient with dextrocardia and unbalanced atrioventricular septal defect, we identified compound heterozygous missense variants in *DNAH9*, a gene recently associated with laterality defects.^22^ Both variants p.(Asp1474Gly) and (Ile4415Thr) were rare and predicted to be damaging. Though pathogenicity could not be established from a molecular perspective, the variants were considered causative due to mild respiratory issues in the proband, and ultrastructural ciliary abnormalities on electron microscopy of nasal mucosa performed after clinical reassessment.

#### Structural variants

Genome sequencing analyses identified two pathogenic CNVs: A 138-kb deletion, including *NOTCH1*, was identified in a proband with TOF and pulmonary atresia, but no obvious other features of Adams–Oliver syndrome. Cosegregation studies in three family members with VSD or TOF could not be performed. A patient with interrupted aortic arch, large VSD, short stature, and dysplastic ears was found to have a de novo 4.1-Mb deletion including *SALL1*, causing Townes–Brocks syndrome.

### Variants of uncertain relevance

Variants that did not meet criteria for pathogenicity were identified in additional families. A family with hypertrophic cardiomyopathy had a predicted damaging, cosegregating missense substitution p.(Gly2080Arg) in *FLNC*, located in a previously disease-associated transmembrane domain.^23^ A patient with dextrocardia and a complex heart defect, short stature, and failure to thrive was identified with rare, compound heterozygous missense variants p.(Thr331Ala) and (Phe3591Leu) in *DNAH8*, a gene associated with primary ciliary dyskinesia. This proband also had a maternally inherited 8.3-Mb deletion at 3p11.2-3p12.3 (including *ROBO1*, a candidate gene for TOF and septal defects^24^). A paternally inherited 594-kb microdeletion at 2p13.1-2p12, encompassing 21 coding genes, was found in a patient with aortic coarctation and bicuspid aortic valve, and a patient with hypoplastic right heart, tricuspid valve dysplasia, septal defect, and mild intellectual disability had a maternally inherited structural aberration involving a 4.3-Mb deletion at 3p26.1-3pter, and a 1.8-Mb duplication at 3p26.1. A 2.4-Mb duplication at 15q13.2-15q13.3 in a proband with interrupted aortic arch, aortic stenosis, and VSD was inherited from the healthy father; however, six largely overlapping duplications of 2–2.5 Mb in the DECIPHER database had occurred de novo, indicating a potential disease locus.

For two variants, though they were classified as likely pathogenic, the causal link to the presenting cardiac condition remained uncertain: a de novo 9-kb deletion of the first exon and promoter region of *DSG2* was identified in a 6-year-old proband with atrial septal defect and dilated right ventricle, but was considered a secondary finding. A pathogenic *PTEN* variant p.(Arg15Ser) segregated in a proband with aortic coarctation and his father with bicuspid aortic valve, yet was not deemed causative for the heart defect according to present knowledge. When applying less stringent variant interpretation principles, the yield of potentially relevant variants could become higher (up to 19.8%; 22/111; Table 2).

### Novel candidate genes

We also analyzed the data for biologically plausible novel gene–disease associations (Table 3; supplementary information). In this respect, we and others^15–17^ had recently reported an association of vascular endothelial growth factor (VEGF) signaling gene(s) and TOF, including two novel variants identified in this cohort: a missense change p.(Ala1030Thr) in the protein kinase domain of *KDR*, and a stopgain variant p.(Arg766*) in *IQGAP1*.^15^ The disease relevance of *IQGAP1* was affirmed by an exome sequencing study, which reported two de novo loss-of-function variants in fetuses with TOF or transposition of the great arteries, respectively.^7^ Consistent with our recently published data, we identified an *FGD5* stopgain variant p.(Glu322*) in a proband with critical pulmonary stenosis and dysplastic valve, adding evidence for an involvement of the VEGF signaling pathway in pulmonary valve development. We also identified a de novo stopgain variant p.(Gln24*) in *CDC42BPA* in a proband with sporadic TOF and right aortic arch. The encoded protein has roles in cytoskeletal remodeling and cell migration, and is a binding partner of CDC42, a GTPase essential for VEGF signaling and developmental processes.^25,26^

**Table 3:** Variants in candidate genes.

ID	Phenotype	Gene	Variant	Inheritance	Biological function^b^
001	TOF, PA	*KDR*^a^ (NM_002253.2)	c.3088G>A, p.(Ala1030Thr)	Maternal	VEGF receptor 2, angiogenesis and vascular development
076	TOF, PS, DORV, failure to thrive, esophageal atresia, bilateral iris coloboma	*IQGAP1*^a^ (NM_003870.3)	c.2296C>T, p.(Arg766*)	Paternal	VEGF signaling, endothelial cell migration
026	PS, dysplastic pulmonary valve, ASD, micrognathia	*FGD5* (NM_152536.3)	c.964G>T, p.(Glu322*)	Maternal	VEGF signaling, endothelial cell migration
007	TOF	*CDC42BPA* (NM_014826.4)	c.70C>T, p.(Gln24*)	De novo	Cytoskeletal reorganization and cell migration
029	HLHS (aortic stenosis, mitral stenosis), short stature, microcephaly, seizures, learning disability, hypotonia, hearing loss	*VASP* (NM_003370.3)	c.[461G>A];[551dupC], p.[(Arg154His)];[(Pro185Thrfs*70)]	Maternal; paternal	Mechanotransduction, response to hemodynamic load
055	HLHS (aortic atresia, mitral stenosis)	*TLN2* (NM_015059.2)	c.[6226G>A];[7141C>G], p.[(Glu2076Lys)];[(Pro2381Ala)]	Maternal; paternal	Mechanotransduction, response to hemodynamic load
021	Tricuspid atresia, pulmonary atresia, mild ADHD, depression	*TRPM4* (NM_017636.3)	c.512G>A, p.(Arg171Gln)	De novo or low-level paternal mosaicism	Regulation of murine heart size
103	AVSD, mild IUGR, lumbar scoliosis with an accessory semisegmented hemivertebra, dysmorphic features	*SMARCC1* (NM_003074.3)	c.1844_1845delAAinsTAAG, p.(Lys615Ilefs*49)	Paternal	Subunit of SWI/SNF complex, chromatin remodeling
056	Dilated cardiomyopathy, deceased at 11 years	*TPCN1* (NM_017901.4)	c.596G>A, p.(Arg199Gln)	De novo	Ca2+ permeable ion channel with potential roles in the regulation of cardiac metabolism
089	ccTGA, VSD, PS	*UBXN10* (NM_152376.3)	c.477_481delTGAAG, p.(Ser159Argfs*44); homozygous	Maternal; paternal	Ciliogenesis, laterality defects in zebrafish

*ADHD* attention deficit hyperactivity disorder, *ASD* atrial septal defect, *AVSD* atrioventricular septal defect, *ccTGA* congenitally corrected transposition of the great arteries, *DORV* double outlet right ventricle, *HLHS* hypoplastic left heart syndrome, *IUGR* intrauterine growth restriction, *PA* pulmonary atresia, *PS* pulmonary stenosis, *TOF* tetralogy of Fallot, *VEGF* vascular endothelial growth factor, *VSD* ventricular septal defect.

Variants are heterozygous/compound heterozygous, unless indicated otherwise.

^a^Previously published Reuter et al.^15^

^b^Details in supplementary information.

In two unrelated families with HLHS, we identified compound heterozygous variants in *VASP* or *TLN2*, respectively (Table 3). Both genes are involved in mechanotransduction of developing cardiomyocytes, linking mechanical strain and cardiac remodeling. Two siblings with hypoplastic right heart had an apparently de novo missense variant p.(Arg171Gln) in *TRPM4*, though one sequencing read suggested potential low-level paternal mosaicism (supplementary information). *Trpm4* is involved in the determination of murine heart size, potentially through a regulation of myocyte proliferation during fetal development.^27^ In a patient with atrioventricular septal defect, mild left ventricular hypoplasia, and extracardiac features, we identified a frameshift insertion p.(Lys615Ilefs*49) in *SMARCC1*, a highly constrained gene encoding a core subunit of the SWI/SNF chromatin remodeling complex. The variant was inherited from the father, diagnosed in adulthood with bicuspid aortic valve. Smarcc1 knockdown in zebrafish was associated with variable multiorgan defects, whereby cardiovascular anomalies were the most penetrant feature.^28^ In humans, haploinsufficiency for other SWI/SNF subunits is associated with developmental disorders, including heart defects: *ARID1A*, *ARID1B*, *ARID2*, *SMARCA4*, *SMARCB1*, *SMARCC2*, *SMARCE1*, *ACTL6A*, and *DPF2*.^29^

A *TPCN1* missense variant p.(Arg199Gln) had occurred de novo in a patient with early-onset, devastating dilated cardiomyopathy. *TPCN1* encodes a lysosomal ion channel (i.e., Ca^2+^), and increased expression was associated with dilated cardiomyopathy and heart failure.^30^ A homozygous frameshift deletion p.(Ser159Argfs*44) in *UBXN10* was found in a patient with congenitally corrected transposition of the great arteries, VSD, and pulmonary stenosis. The encoded protein is required for ciliogenesis, and Ubxn10 depletion caused cardiac laterality defects in zebrafish.^31^ Predicted loss-of-function variants in *GMDS* (Ebstein anomaly), *SRPK2* (HLHS), and *TOP2A* (HLHS) were also considered candidates for the cardiac phenotypes (supplementary information).

## DISCUSSION

The value of genetic testing in severe, congenital disorders is widely recognized, as it may specify recurrence risks and potential comorbidities, and will ultimately support optimized clinical management and outcomes.^32^ Implementation of standardized genetic testing protocols in infants with critical CHD had resulted in higher diagnostic rates and cost efficiency.^33^ Nonetheless, there is presently no consensus on appropriate genetic testing in families with cardiac lesions, and the genomic architecture is fairly unknown.

This study set out to investigate the diagnostic utility of genome sequencing in a cohort of pediatric heart disease. Although many of the families had undergone prior genetic testing (Table S1), genome sequencing identified a disease-causing variant in 14 of 111 probands (12.6%). The majority of genes were associated with “syndromic disease” (e.g., *ANKRD11*, *KMT2D*, and *POGZ*) and were detected in individuals with extracardiac features. Particularly in young children, associated features may be nonspecific, or erroneously attributed to the cardiac lesion. A further aspect impeding the recognition of genetic syndromes is the tremendous clinical variation even of well-defined disorders. This was evident as pathogenic alleles were inherited from ostensibly healthy parents (e.g., *MYH11*, *NIPBL*, and *PTPN11*). By contrast, six of seven variants primarily associated with transcriptional regulation/chromatin organization were de novo, potentially due to pleiotropy and a higher rate of extracardiac features^12^ (Fig. S2). Immediate implications on patient management and genetic counseling were related to variants in *ANKRD11*, *DNAH9*, *DSG2*, *KMT2D*, *MYH11*, *NEXMIF*, *NIPBL*, *NOTCH1*, *PURA*, *POGZ*, *PTEN*, *PTPN11* (×2), and *SALL1*.

The variety of clinically unexpected—or formerly undetected—findings supports a role of genome sequencing as a first-tier diagnostic test in patients with CHD.^34^ Genome sequencing was previously shown to have adequate coverage for clinically relevant gene sets,^9^ and can overcome several technical limitations of exome sequencing and chromosomal microarray analysis, particularly for small structural variations.^35^ In this study, we demonstrate the detection and fine-mapping of a wide size range of potentially disease-related CNVs (9.1 kb to 8.3 Mb), with reliable detection rates previously shown to exceed microarrays.^36^

For the majority of individuals in this study, the etiology of their heart defects remained unknown. This held true even for some families where a genetic condition was strongly suspected. Though genome sequencing has the potential to capture most types of interindividual genomic differences, our abilities to identify those that are disease-relevant lag behind. This particularly applies for noncoding regions and synonymous variants, but also for the multitude of nonsynonymous alterations with uncertain effects upon protein function. Genomic curation largely depends on manual data review, even when applying assisting software to partially automate the process.^37^ Variant interpretation in congenital heart disease can be particularly challenging due to limited genotype–phenotype correlations and incomplete penetrance. Disease associations and functional studies from the literature need to be critically reviewed and potentially reassessed. Even when applying established guidelines,^10^ the evaluation of genomic variation is subjective and potentially discordant among analysts.^38^ With stringent application of the ACMG guidelines, we consider our interpretation to be conservative, compared with CHD studies with higher diagnostic yields^5,8^ (Table S3). As for other heterogeneous diseases with incomplete penetrance, the contribution of rare, inherited variants is most likely underestimated. A comprehensive delineation of the genomic spectrum will involve statistical approaches and functional assays. The full potential of genomic data analysis will evolve prospectively, and the yield is expected to increase accordingly.

By study design, findings from this cohort were not transferable to a general CHD population. We enriched the cohort for more complex cardiac conditions with a supposedly stronger genetic etiology, such as outflow tract anomalies and single functional ventricles,^4^ though we also identified relevant diagnoses in families with isolated lesions. On the other hand, many probands had negative clinical genetic testing prior to study enrollment (Table S1), which may account for the relatively low numbers of pathogenic CNVs, for instance.^39^ In this study, the outcome of the testing was associated with the presence of extracardiac features, but not a positive family history for clinically relevant CHD. As other data suggested the diagnostic yield to also depend on the fraction of familial cases,^5^ larger studies will need to refine which patients will most likely benefit from genomic testing.

In the attempt to disentangle the genetic basis of pediatric heart disease, our systematic analysis revealed possible new candidate genes (Table 3). We prioritized variants based on known disease mechanisms in cardiac development, such as disruptions of critical biological functions and pathways, or altered dosage of constrained signaling genes.^3^ However, the validation of novel gene–disease associations is challenged by the genetic heterogeneity, as recurrence in unrelated families, or ideally significant enrichment on a variant or gene level, would require very large cohorts. Small pedigrees and incomplete penetrance (e.g., through multilocus inheritance with rare and common modifiers^40^) further impede classical linkage or cosegregation evidence. The functional assessment of candidate genes in animal or cellular models is time-consuming, and transferability to human heart development is limited. Sharing potential (unverified) gene–disease associations, e.g., in databases and the scientific literature, is therefore evidentially valuable for the assembly of independent evidence and the design of follow-up studies.

Our data outline the diagnostic and scientific utility of comprehensive (nontargeted) genetic testing in families with pediatric heart disease, and anticipate that genome sequencing will ultimately become a first-tier diagnostic test. Many cardiac disease–gene associations are likely yet to be unraveled, and this attempt will require large-scale genomic initiatives and interdisciplinary efforts for experimental validations.

## Supplementary information

Supplementary Material

Supplementary Table S1
