# Supplementary material for: The Cardiac Genome Clinic: implementing genome sequencing in pediatric heart disease
Source: Genet Med. 2020 Feb 10;22(6):1015–24. doi: 10.1038/s41436-020-0757-x (PMC7272322; doi:10.1038/s41436-020-0757-x)
Supplement: Supplementary file 1 — Supplementary Material [file 41436_2020_757_MOESM1_ESM.docx]

**Supplementary material for:**

**The Cardiac Genome Clinic: Implementing genome sequencing in pediatric heart disease**

Miriam S Reuter, Rajiv R Chaturvedi, Eriskay Liston, Roozbeh Manshaei, Ritu B Aul, Sarah Bowdin, Iris Cohn, Meredith Curtis, Priya Dhir, Robin Hayeems, S Mohsen Hosseini, Reem Khan, Linh G Ly, Christian R Marshall, Luc Mertens, John BA Okello, Sergio L Pereira, Akshaya Raajkumar, Mike Seed, Bhooma Thiruvahindrapuram, Stephen W Scherer, Raymond H Kim, Rebekah Jobling

**Supplementary information - Study participants**

We recruited 111 families with one or more affected individuals with congenital heart defects or early-onset cardiomyopathies (recruitment through the Ted Rogers Cardiac Genome Clinic, from 01-2017 to 12-2018, at the Hospital for Sick Children). Details on phenotypes, family history, prior assessments, and sequencing strategies were entered into PhenoTips^[1](#_ENREF_1)^ (Table S1).

**Table S1. Study participants and variants of uncertain significance.**

[Provided as a separate table]

Abbreviations: ADHD, attention deficit hyperactivity disorder; ASD, atrial septal defect; AVSD, atrioventricular septal defect; BAV, bicuspid aortic valve; CoArc, aortic coarctation; DORV, double outlet right ventricle; HCM, hypertrophic cardiomyopathy; HLHS, hypoplastic left heart; ID, intellectual disability; IUGR, intrauterine growth retardation; IVC, inferior vena cava; IVS, intact ventricular septum; LQTS, long QT syndrome; MA, mitral atresia; NA, not applicable; OCD, obsessive compulsive disorder; PA, pulmonary atresia; PDA, patent ductus arteriosus; PS, pulmonary stenosis; SVC, superior vena cava; SVT, supraventricular tachycardia; TAPVR, total anomalous pulmonary venous return; TGA, transposition of the great arteries; TOF, tetralogy of Fallot; VSD, ventricular septal defect.


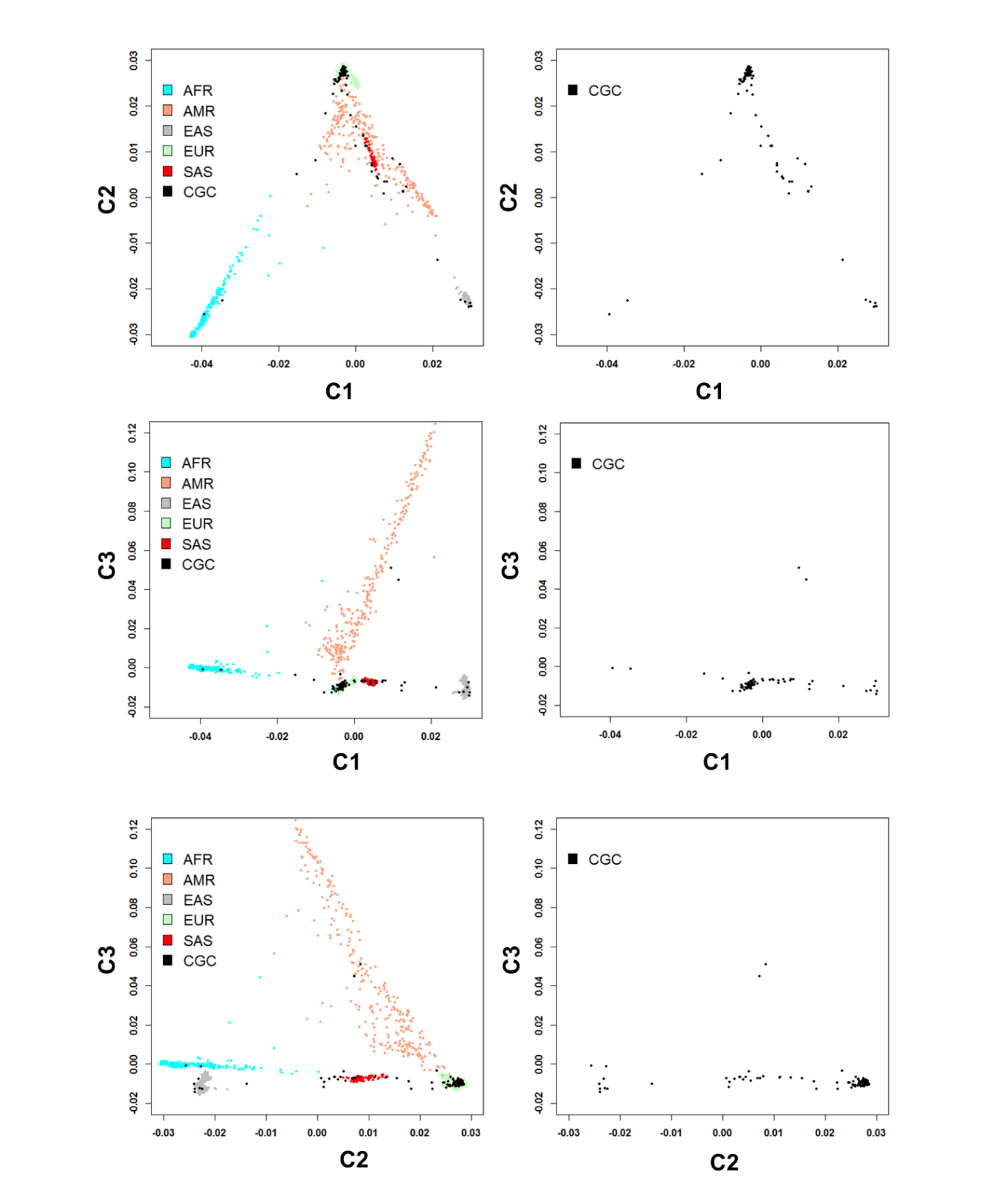


**Figure S1. Principle component analysis of 111 probands with CHD.** Principal component analysis to detect population structure for the 1000 Genomes Project and Cardiac Genome Clinic samples. Each individual is represented by a dot. The 1000 Genomes Project samples are colored by the continental groups and the CGC samples are coloured in black.

Abbreviations: AFR, African; AMR, American; CGC, Cardiac Genome Clinic; EAS, East Asian; EUR, European; SAS, South Asian.

**Supplementary methods - Genome sequencing**

DNA from whole blood (n=328) was sequenced on the Illumina HiSeq X system at The Centre for Applied Genomics (TCAG) in Toronto, Canada. DNA was quantified using the Qubit High Sensitivity Assay, and sample purity was checked using the Nanodrop OD260/280 ratio. Following the manufacturer’s recommended protocol, 700 ng of genomic DNA were used as input material for library preparation using the Illumina TruSeq PCR-free DNA Library Prep Kit. In brief, DNA was fragmented to an average of 400 bp by sonication on a Covaris LE220 instrument. Fragmented DNA was end-repaired and A-tailed and indexed TruSeq Illumina adapters added by ligation. Libraries were assessed using Bioanalyzer DNA High Sensitivity (Agilent) for sizing and absence of primer dimers, and quantified by quantitative PCR using Kapa Library Quantification Illumina/ABI Prism Kit protocol (KAPA Biosystems). Each validated libraries were sequenced in one lane on an Illumina HiSeq X platform following Illumina’s recommended protocol to generate paired-end reads of 150 bases in length.

**Variant calling and annotation**

Base calling was performed using BCL2FASTQ, and data were generated using Illumina HiSeq Analysis Software (HAS; version 2-2.5.55.1311). Reads were mapped to the GRCh37/hg19 reference sequence, using the BWA-backtrack algorithm from BWA v0.7.12 or v0.7.15 [^2^](#_ENREF_2). Small nucleotide variants (SNVs) and insertions/deletions (indels) were called using GATK v3.5 or v3.7, according to GATK Best Practices recommendations [^3^](#_ENREF_3)^,^ [^4^](#_ENREF_4). Copy number variants (CNVs) were called using a modified read depth method with the programs ERDS v1.1 (Estimation by Read Depth with Single-nucleotide variants) [^5^](#_ENREF_5) and CNVnator v0.3.3 [^6^](#_ENREF_6) using a window size of 500 bp. CNV size cut-offs were 1 kb for losses and 2 kb for gains. High-quality CNVs were defined as those detected by ERDS that were also detected by CNVnator with greater than 50% overlap [^7^](#_ENREF_7). Structural variants (SVs: deletions, duplications, insertions and inversions) were detected using the algorithm Manta [^8^](#_ENREF_8). Variant calls were annotated using a custom pipeline developed at TCAG based on ANNOVAR [^9^](#_ENREF_9).

**Variant assessment**

1. Predicted loss-of-function alleles

We predicted the following as loss-of-function (LoF) or null alleles: frameshift insertions, deletions or substitutions; substitutions creating a stop codon; and alterations of the intronic dinucleotide adjacent to a coding-exonic splice junction.

1. Allele frequency and control databases

Overall and population-specific allele frequencies for SNVs and small indels were derived from 1000 Genomes (African, American, East Asian, European, South Asian; http://www.internationalgenome.org/), ExAC (African, American, East Asian, Finnish, Non-Finnish Europeans, South Asians, Others; http://exac.broadinstitute.org/) [^10^](#_ENREF_10), and gnomAD (African, American, Ashkenazi Jewish, East Asian, Finnish, Non-Finnish Europeans, South Asians, Others; http://gnomad.broadinstitute.org/) [^11^](#_ENREF_11). For CNVs and other SVs, allele frequencies were compared to unaffected parents in the Autism Speaks MSSNG dataset [^12^](#_ENREF_12). The Database of Genomic Variants (http://dgv.tcag.ca) was also used to adjudicate CNVs [^13^](#_ENREF_13).

1. Disease variant databases

DECIPHER (https://decipher.sanger.ac.uk/), the Human Gene Mutation Database (HGMD; http://www.hgmd.cf.ac.uk/ac/index.php) and ClinVar (https://www.ncbi.nlm.nih.gov/clinvar/) were used as disease variant databases.

1. Validation of variants

Read alignments for all putative disease-associated variants were manually inspected using IGV (http://software.broadinstitute.org/software/igv/). All variants described in Tables 2 and 3 were confirmed by clinical testing or research Sanger sequencing (primers and sequencing protocols are available upon request). The confirmation rate in probands was 100%. Co-segregation studies were performed in available family members.

**Statistics**

We performed Fisher’s exact test for count data in R version 3.4.4.

**Table S2. Genome sequencing coverage statistics.**

| **Sequenced individuals (n=328)** | **Mean coverage per genome** | **Proportion with coverage 10x or greater** |
| --- | --- | --- |
| **Average** | 36.6 | 0.97 |
| **Median** | 36.7 | 0.97 |
| **Range** | 25.6 - 50.6 | 0.96 - 0.98 |

**Supplementary results - Families with variants of interest**

**(Additional variants of uncertain significance in Table S1)**

Family 001 (same family as in [^14^](#_ENREF_14)) :

Mother and daughter with tetralogy of Fallot (TOF) and pulmonary atresia. The mother also had mild intellectual disability. A cousin with total anomalous pulmonary venous return was also genome sequenced.

| ***KDR* (NM_002253.2)** | **c.3088G>A, p.(Ala1030Thr)** | **chr4:55956227C>T** |
| --- | --- | --- |

We recently associated loss-of-function variants in *KDR* with tetralogy of Fallot (**candidate gene of uncertain significance**) [^15^](#_ENREF_15). *KDR* encodes vascular endothelial growth factor receptor 2 (VEGFR2), which has pivotal roles in angiogenesis and vascular development. This missense variant was identified in a mother and daughter with TOF, but not a cousin with total anomalous pulmonary venous return. The *KDR* missense variant was not reported in controls (MAF=0) and substitutes an amino acid in the protein kinase domain, adjacent to the catalytic residues Asp1028 and Arg1032 [^16^](#_ENREF_16). The variant was *in silico* predicted to be damaging (SIFT=0, PolyPhen2=1, CADD=35).

Family 007:

Proband with sporadic TOF, right aortic arch, and no extracardiac anomalies.

| ***CDC42BPA* (NM_014826.4)** | **c.70C>T, p.(Gln24*)** | **chr1:227504814G>A** |
| --- | --- | --- |

We identified a *de novo* variant in *CDC42BPA*, a **candidate gene of uncertain significance**. *CDC42BPA* is constrained (pLI=0.99) and encodes a protein kinase, with functions in cytoskeletal reorganization and cell migration [^17^](#_ENREF_17)^,^ [^18^](#_ENREF_18). It was a binding partner of CDC42, which had important functions in vascular endothelial growth factor (VEGF) signaling and was associated with diverse developmental phenotypes, including heart defects [^19^](#_ENREF_19)^,^ [^20^](#_ENREF_20). The variant was not reported in controls (MAF=0).

Family 010:

2 brothers and father with hypertrophic cardiomyopathy.

| ***FLNC* (NM_001458.4)** | **c.6238G>C, p.(Gly2080Arg)** | **chr7:128493552G>C** |
| --- | --- | --- |

Missense variants in *FLNC* were associated with hypertrophic cardiomyopathy [^21^](#_ENREF_21). This **variant of uncertain significance** (PM1, PM2, PP3) segregated with the disease in all three affected individuals. The variant was not reported in controls (MAF=0). *In silico* tools predicted it to be damaging (SIFT=0, PolyPhen2=1, CADD=27.8). The affected domain (Ig-like domain 19) was enriched for disease-associated variation [^22^](#_ENREF_22)^,^ [^23^](#_ENREF_23).

Family 013:

Proband with small muscular ventricular septal defect (VSD), axial hypotonia, hypoventilation, infantile spasms, macrosomia, macrocephaly, and global developmental delay.

| ***PURA* (NM_005859.4)** | **c.812_814delTCT,**  **p.(Phe271del)** | **chr5:139494578_139494580delTCT** |
| --- | --- | --- |
| ***PTEN* (NM_000314.4)** | **c.395G>A, p.(Gly132Asp)** | **chr10:89692911G>A** |

Both variants occurred *de novo*, and were recurrent, **pathogenic** variants (*PURA*: [^24^](#_ENREF_24)^,^ [^25^](#_ENREF_25) (PS2, PS4, PM2); *PTEN*: [^26-31^](#_ENREF_26) (PS2, PS4, PM1, PM2)). None of the two variants was reported in controls (MAF=0). Both defects likely contributed to the phenotype, and a minority of patients with PURA-related disorders also presented with structural heart defects [^32^](#_ENREF_32).

Family 018:

Proband with dysplastic aortic and pulmonary valve, pulmonary stenosis, aortic regurgitation, and borderline short stature (at the age of 5 months).

| ***PTPN11* (NM_002834.4)** | **c.209A>G, p.(Lys70Arg)** | **chr12:112888193A>G** |
| --- | --- | --- |

We identified a recurrent **likely pathogenic** *PTPN11* variant (Noonan syndrome; [^33^](#_ENREF_33); PS4, PM1, PM2). The variant was inherited from the mother (MAF=0), who was considered healthy but had decreased ventricular function revealed by the research echocardiography.

| ***NOTCH1* (NM_017617.5)** | **c.1934G>A, p.(Cys645Tyr)** | **chr9:139409822C>T** |
| --- | --- | --- |

The *NOTCH1* variant was recently reported in a patient with nonsyndromic tetralogy of Fallot [^34^](#_ENREF_34), and was a maternally inherited **variant of uncertain significance** (PM2, PP3). *In silico* tools predicted the variant to be damaging (SIFT=0, PolyPhen2=1, CADD=29). MAF=0

Family 019:

Proband with interrupted aortic arch (B-type), aortic valvular stenosis, small aortic valve, hypoplastic ascending aorta, small perimembranous VSD, small atrial septal defect (ASD), apnea, intraventricular hemorrhage (after premature birth at 28 weeks), microcephaly, dysmorphisms, anemia, and hyponatremia (deceased at 2 months).

| **15q13.2-15q13.3** | **Duplication (2.4 Mb)** | **chr15:30388001-32766000** |
| --- | --- | --- |

We identified a paternally inherited **copy number variant of unknown significance**. Overlapping duplications were reported in the DECIPHER database, in patients with developmental delay, intellectual disability, and some with congenital heart defects. Sixteen largely overlapping copy number gains were of similar size (2-2.5Mb): 6 of them were *de novo*, 7 of unknown inheritance, and 3 inherited.

Family 021:

Proband with hypoplastic right heart, tricuspid atresia, pulmonary atresia, a very small ventricular septal defect, mild attention deficit hyperactivity disorder (ADHD), and depression. Her brother died in utero with hypoplastic right heart, small tricuspid valve, large VSD, small aorta arising from the right ventricle (double outlet right ventricle).

| ***TRPM4* (NM_017636.3)** | **c.512G>A, p.(Arg171Gln)** | **chr19:49671580G>A** |
| --- | --- | --- |

Both affected siblings had a missense variant in *TRPM4* (**candidate gene of uncertain significance**; MAF=0). The variant was *de novo* or low-level mosaic in one parent (likely the father, as the A-allele was present in 1/48 sequencing reads). TRPMs are Ca^2+^-permeable cation channels localized predominantly within the plasma membrane. *TRPM4* gain-of-function variants were associated with heart block and ECG anomalies (OMIM-P # 604559). The encoded protein was involved in the determination of murine heart size (potentially through negative regulation of cardiomyocyte proliferation during prenatal development; [^35^](#_ENREF_35)). Higher gene expression was associated with physiological cardiac hypertrophy in mice/rats [^36-38^](#_ENREF_36). TRPM4 was also associated with mechanosensation/transduction in vascular smooth muscle cells [^39-41^](#_ENREF_39).

Family 026:

Proband with critical pulmonary stenosis and micrognathia.

| ***FGD5* (NM_152536.3)** | **c.964G>T, p.(Glu322*)** | **chr3:14861542G>T** |
| --- | --- | --- |

We recently associated loss-of-function variants in *FGD5* with tetralogy of Fallot (**candidate gene of uncertain significance**) [^14^](#_ENREF_14). *FGD5* is constrained (pLI=1), and the encoded protein was involved in VEGF downstream signaling and inhibition of VEGFR2 degradation [^42^](#_ENREF_42)^,^ [^43^](#_ENREF_43). The variant was inherited from the healthy mother and was not reported in controls (MAF=0).

Family 027:

Proband with aortic stenosis, dysplastic aortic valve, global developmental delay, self injurious behaviour, microcephaly, short stature, hypotonia, joint laxity, and dysmorphic features. The mother had mild intellectual disability and epilepsy.

| ***NEXMIF* (NM_001008537.2)** | **c.1502delG, p.(Gly501Valfs*4)** | **chrX:73962890delC** |
| --- | --- | --- |

**Likely pathogenic** *NEXMIF* variant (PVS1, PM2), which explained the neurodevelopmental phenotype. CHD was not a common feature of NEXMIF-associated disease, but valve dysfunctions (pulmonary stenosis, mitral insufficiency) was infrequently reported, indicating a potential role of the gene in valve development [^44^](#_ENREF_44)^,^ [^45^](#_ENREF_45).

Family 029:

Proband with hypoplastic left heart syndrome, short stature, microcephaly, seizures, learning disability, hypotonia, hearing loss, leg length discrepancy, and osteopenia.

| ***VASP* (NM_003370.3)** | **c.[461G>A];[551dupC],**  **p.[(Arg154His)];[(Pro185Thrfs*70)]** | **chr19:46025458G>A,**  **chr19:46025663dupC** |
| --- | --- | --- |

Compound heterozygous variants in *VASP* (**candidate gene of uncertain significance**): Variant 1 was a frameshift duplication, variant 2 a missense variant close to a phosphokinase A phosphorylation site (Ser157 [^46^](#_ENREF_46)), which was predicted to be damaging (SIFT=0, PolyPhen2=1, CADD=25.5). *VASP* encodes an actin remodeling protein in cardiomyocytes [^47^](#_ENREF_47). VASP was presumed to encode a modifier, affecting the response of heart to hemodynamic overload or other stress conditions, and potentially required for a correct hypertrophy response when the heart was subjected to hemodynamic overload [^48^](#_ENREF_48). VASP phosphorylation was associated with response to volume overload in canine hearts [^49^](#_ENREF_49), vasodilation and angiogenesis [^50^](#_ENREF_50)^,^ [^51^](#_ENREF_51). MAF=0.0001 / 0.00009.

Family 032:

Proband with unbalanced atrioventricular septal defect (AVSD) committed to the left ventricle, hypoplastic right ventricle, common atrioventricular valve, large primum ASD, small inlet VSD, borderline microcephaly, global developmental delay, hypotonia, and gastro-jejunal tube feeding. Mother with short stature, brachydactyly, delayed menarche, but otherwise healthy and no cardiac lesions or cognitive deficits.

| ***NIPBL* (NM_133433.3)** | **c.771+1G>A, p.?** | **chr5:36971139G>A** |
| --- | --- | --- |

Maternally inherited, recurrent **pathogenic** variant for Cornelia de Lange syndrome (PVS1, PS4, PM2, de novo in [^52^](#_ENREF_52)). The variant was not reported in controls (MAF=0).

Family 033:

Proband with TOF, pulmonary atresia, and hypoplastic pulmonary arteries. Several family members were reported with CHD: the father and paternal grandmother had a ventricular septal defect, the grandmother's deceased sister had TOF.

| ***NOTCH1*, 9q34.3** | **Deletion (138 kb)** | **chr9:139345895-139484281del** |
| --- | --- | --- |

**Likely pathogenic** *NOTCH1* deletion (PVS1, PM2). *NOTCH1* was associated with Adams-Oliver syndrome or nonsyndromic CHD, e.g., TOF [^34^](#_ENREF_34). Other genes within the deletion were *SEC16A*, *C9orf163*. Inheritance of the deletion could not be assessed, but the family history indicated that it was likely paternally inherited.

Family 034 (same family as in [^14^](#_ENREF_14)):

Proband with TOF, pulmonary atresia with major aortopulmonary collateral arteries, non-confluent hypoplastic pulmonary arteries, and congenital lymphedema. Mother had a normal research echocardiography.

| ***FLT4* (NM_182925.4)** | **c.89delC, p.(Pro30Argfs*3)** | **chr5:180058748delG** |
| --- | --- | --- |

*FLT4* haploinsufficiency was recently associated with incompletely penetrant, nonsyndromic tetralogy of Fallot [^14^](#_ENREF_14)^,^ [^34^](#_ENREF_34)^,^ [^53^](#_ENREF_53). This **likely pathogenic** (PVS1, PM2), recurrent variant [^53^](#_ENREF_53) was inherited from the healthy mother.

Family 039:

Proband with aortic coarctation, transverse arch hypoplasia, bicuspid aortic valve (BAV), small anteriormuscular VSD, macrocephaly, hemangioma, scoliosis, intellectual disability.

| ***NR2F2* (NM_021005.3)** | **c.671T>A, p.(Val224Asp)** | **chr15:96877533T>A** |
| --- | --- | --- |

*De novo*, **likely pathogenic** variant in *NR2F2* (PS2, PM2, PP3). *De novo* and segregating *NR2F2* missense variants were identified in individuals with congenital heart defects (particularly septal defects), who had a broad phenotypic spectrum of associated extracardiac findings, inclunding developmental delay, autistic behaviour, diaphragmatic hernia, limb anomalies, and dysmorphic features [^54-56^](#_ENREF_54). MAF=0

Family 042:

Proband with balanced AVSD, large primum and secundum ASD, inlet VSD, severe left pulmonary vein stenosis, feeding difficulties, polyhydramnion, dilated bowel, renal cyst, choroid plexus cyst, micrognathia (deceased at the age of 7 months).

| ***ANKRD11* (NM_013275.5)** | **c.5238_5239delGC,**  **p.(Pro1747Argfs*49)** | **chr16:89347711delGC** |
| --- | --- | --- |

*De novo* **pathogenic** variant for KBG syndrome (PVS1, PS2, PM2): 10-26% of KBGs syndrome had cardiac defects, including ASD and VSD [^57^](#_ENREF_57). MAF=0

Family 043:

Proband with severe Ebstein's anomaly, oligohydramnion, pulmonary hypoplasia, short stature, microcephaly, rhizomelia, facial dysmorphisms, hypotonia, and gross motor delay.

| ***GMDS*, 6p25.3** | **Deletion (146 kb)** | **chr6:2105243-2251273del** |
| --- | --- | --- |

Maternally inherited *GMDS* deletion (**candidate gene of** **uncertain significance**; pLI=0.99). *GMDS* was reported as a candidate for CHD, intellectual disability, short stature, microcephaly, and facial dysmorphisms, based on the smallest overlapping region in 6p25 microdeletion syndromes and the constraint metrics of the gene. However, 6p25 microdeletion was associated with extreme inter-individual variability in clinical phenotypes [^58^](#_ENREF_58)^,^ [^59^](#_ENREF_59). The encoded protein catalyzed the first step in protein fucosylation, which is an essential posttranslational modification in members of the Notch family of trans-membrane receptors [^58^](#_ENREF_58)^,^ [^60^](#_ENREF_60)^,^ [^61^](#_ENREF_61).

Family 044:

Proband with aortic coarctation, tubular hypoplasia, multiple apical VSD, fenestrated ASD, functional BAV, mild developmental delay, head circumference at the 87th centile, and hearing loss. The father had BAV.

| ***PTEN* (NM_000314.6)** | **c.45A>C, p.(Arg15Ser)** | **chr10:89624271A>C** |
| --- | --- | --- |

Paternally inherited pathogenic variant of **uncertain disease contribution** (PS1, PS3, PS4, PP5, PP2, PP3). The same amino acid substitution was reported to be pathogenic in germline ([^62^](#_ENREF_62): macrocephaly, GI hamartomas; [^63^](#_ENREF_63): CS-like; [^64^](#_ENREF_64)) and cancer (COSM5270). The variant affected a conserved amino acid, which was located within a cluster of LP/P variants in the phosphatase tensin-type domain (UniProt). Reduced PTEN activity was demonstrated by multiple functional assays [^65-67^](#_ENREF_65), and the mutant protein was unable to suppress cell proliferation [^68^](#_ENREF_68). Although heart defects were not considered typical for PTEN hamartoma syndrome, vascular anomalies of diverse organs were commonly found [^69^](#_ENREF_69), and PTEN was associated with regulation of the PI3K/AKT pathway and cardiovascular morphogenesis [^70^](#_ENREF_70). The variant was absent in controls (MAF=0).

Family 051:

Proband with dextrocardia, mirror image atrial arrangement, transposition of the great arteries (TGA), bilateral superior venae cavae (left SVC to left-sided atrium, right SVC to coronary sinus), and situs inversus totalis: left-sided liver, right-sided spleen.

| ***PKD1L1* (NM_138295.4)** | **c.6473+2_6473+3delTG, p.?** | **chr7:47870812delCA** |
| --- | --- | --- |

One pathogenic variant of **uncertain disease contribution** (PVS1, PM2, PP5). A second likely pathogenic variant in *PKD1L1* was not identified.

Family 054:

Proband with patent ductus arteriosus (PDA). One paternal uncle, one aunt, and two cousins also had PDA. The paternal grandfather had died of an aortic aneurysm in his 80ies.

| ***MYH11* (NM_002474.2)** | **c.4578+1G>A, p.?** | **chr16:15815278C>T** |
| --- | --- | --- |

Paternally inherited **pathogenic** variant (PS1, PS3, PM2, PM4). The same variant was reported in a family with thoracic aortic aneurysm and dissection (TAAD) and PDA [^71^](#_ENREF_71) and a patient with aortopathy [^72^](#_ENREF_72). Other variants affecting the same nucleotide or the same canonical splice site were also reported to be pathogenic for TAAD and PDA [^73^](#_ENREF_73), and in a family with PDA [^74^](#_ENREF_74). c.4578+1G>T was shown to cause an in-frame deletion/loss of exon 32 (71 amino acids) [^73^](#_ENREF_73). The variant was not reported in controls (MAF=0).

Family 055:

Proband with hypoplastic left heart (HLHS), mitral stenosis, aortic atresia, and coronary sinusoids and fistula.

| ***TLN2* (NM_015059.2)** | **c.[6226G>A];[7141C>G],**  **p.[(Glu2076Lys)];[(Pro2381Ala)]** | **chr15:63089593G>A;**  **chr15:63127948C>G** |
| --- | --- | --- |

Two rare, compound heterozygous variants in a **candidate** **gene of uncertain significance** (MAF=0 / 1.22E-05). The encoded protein was highly expressed in heart, brain, and skeletal muscle [^75^](#_ENREF_75), and was involved in mechanotransduction and cardiac remodeling in response to hemodynamic overload by physically linking integrin to the actin cytoskeleton [^75-77^](#_ENREF_75). Talin2 regulated traction force generation and focal adhesion dynamics, thus regulating cardiovascular integrity [^78^](#_ENREF_78).

Family 056:

Proband with early-onset dilated cardiomyopathy, abnormal skin blistering, and poor wound healing (deceased at 11 years).

| ***TPCN1* (NM_017901.4)** | **c.596G>A, p.(Arg199Gln)** | **chr12:113706614G>A** |
| --- | --- | --- |

A *de novo* variant in a **candidate gene of uncertain significance**. The variant was not reported in controls, and predicted to be damaging (SIFT=0.00, PolyPhen2=0.99, CADD=34). *TPCN1* encodes a voltage-gated ion channel expressed exclusively in acidic organelles such as endosomes and lysosomes, permeable to Ca2+ and monovalent cations [^79^](#_ENREF_79), and also responsible for NAADP-mediated Ca2+ release [^80^](#_ENREF_80)^,^ [^81^](#_ENREF_81). This group of proteins (TPCNs) had potential roles in the regulation of cardiac metabolism [^82^](#_ENREF_82), including autophagy [^83^](#_ENREF_83), Ca2+ oscillations, and cell death [^84^](#_ENREF_84). Specifically, increased *TPCN1* expression was associated with DCMP and heart failure [^85^](#_ENREF_85). This specific variant p.(Arg199Gln) was previously modelled in the mouse homologue Tpc1 (Arg200Gln) and overexpressed in human embryonic kidney cells. Although the missense substitution did not affect voltage-gating properties of the channel, other changes in functions of the protein were not investigated [^86^](#_ENREF_86).

Family 058:

Proband with HLHS, mitral atresia, double outlet right ventricle (DORV), VSD, hypoplastic aortic arch, coarctation, short stature, microcephaly, retrognathia, and clinodactyly.

| ***SRPK2*, 7q22.3** | **Intragenic duplication (203 kb)** | **chr7:104775001-104978000dup** |
| --- | --- | --- |

*De novo* intragenic tandem duplication, predicted to cause a heterozygous loss of SRPK2 function. *SRPK2* is a constrained **candidate gene of uncertain significance** (pLI=1). Its absence led to massive genomic instability revealed by high levels of DNA double-strand breaks [^87^](#_ENREF_87). SRPK1/2 inhibition blocked the splicing of a pro-angiogenic VEGFA isoform, and resulted in reduced angiogenesis in a mouse model of retinal neovascularization [^88^](#_ENREF_88). The gene was expressed in brain, heart and skeletal muscle [^89^](#_ENREF_89).

Family 059:

Proband with ASD secundum and moderately dilated right ventricle. His brother, mother and potentially a paternal uncle also had ASD.

| ***DSG2*, 18q12.1** | **Deletion (9.1kb)** | **chr18:29073480-29082599del** |
| --- | --- | --- |

The proband had a *de novo* deletion of the first exon and promoter region of *DSG2*. The variant was **likely pathogenic** (PVS1, PM2) for arrhythmogenic right ventricular cardiomyopathy, which might be causative for the right ventricular dilation reported in the proband. An association with atrial septal defects was not reported, and the variant did not segregate with this phenotype in the family.

Family 060:

Proband with aortic coarctation, transverse arch hypoplasia, bovine arch (common origin brachiocephalic and left common carotid arteries), VSD (doubly committed subarterial VSD), posterior deviation of the outlet septum, tri-leaflet aortic valve, subaortic ridge, prenatally increased nuchal translucency, short stature, failure to thrive, facial dysmorphisms, ptosis, joint hypermobility/hypotonia, and cryptorchidism.

| ***PTPN11* (NM_002834.4)** | **c.923A>G, p.(Asn308Ser)** | **chr12:112915524A>G** |
| --- | --- | --- |

Known **pathogenic** *de novo* variant (PS1, PS2, PS3, PS4, PM2, PP2; this or other nucleotide substitutions affecting the same amino acid were reported in multiple patients with Noonan syndrome). The variant was not reported in controls (MAF=0). Functional studies suggested altered substrate specificity at the catalytic site [^90^](#_ENREF_90). The proband’s variant was also identified by a clinical panel, performed in parallel (and independently) to the genome sequencing analysis.

Family 074:

Proband with interrupted aortic arch type B, hypoplastic ascending aorta and transverse arch, large VSD (outlet septum to mid-muscular septum), posterior deviation of the outlet septum, left SVC to coronary sinus, borderline short stature, hearing impairment, and dysplastic ears with preauricular tags.

| **6q12.1-16q12.2 (incl. *SALL1*)** | **Deletion (4.1 Mb)** | **chr16:49326510-53476612del** |
| --- | --- | --- |

**Pathogenic** *de novo* deletion of *SALL1* (Towns-Brock syndrome) and other genes: *C16orf78*, *ZNF423*, *CNEP1R1*, *HEATR3*, *TENT4B*, *ADCY7*, *BRD7*, *NKD1*, *SNX20*, *NOD2*, *CYLD*, *C16orf97*, *TOX3*, *CHD9*, *LOC643802*, *RBL2*. *SALL1* haploinsufficiency was likely causative for the majority of clinical features (OMIM-P 107480), but other genes may contribute. The deletion was in parallel (and independently) identified by clinical microarray.

Family 076 (same family as in [Reuter et al.]:

Proband with TOF with pulmonary stenosis and DORV, confluent pulmonary arteries, left SVC to coronary sinus, no bridging vein, left aortic arch, esophageal atresia, bilateral iris coloboma, and failure to thrive.

| ***IQGAP1* (NM_003870.3)** | **c.2296C>T, p.(Arg766*)** | **chr15:91016189C>T** |
| --- | --- | --- |

Rare, paternally inherited loss-of-function variant in *IQGAP1* (MAF=0.000004). We recently reported this constrained **candidate gene of uncertain significance** for tetralogy of Fallot [^14^](#_ENREF_14). Independently, two *de novo* loss-of-function variants were recently reported in fetuses with TOF or transposition of the great arteries, respectively [^91^](#_ENREF_91). *IQGAP1* encodes a GTPase-binding scaffold protein regulated by VEGFR2 signaling, with roles in angiogenesis, endothelial cell polarization, migration and proliferation [^92^](#_ENREF_92)^,^ [^93^](#_ENREF_93).

Family 081:

Proband with HLHS, mitral stenosis aortic atresia, ADHD, nephrocalcinosis, hypercalcemia, dysmorphisms (parietal bossing, broad forehead, and preauricular pit).

| ***DNAH11* (NM_001277115.1)** | **c.[3494A>G]; [9952C>T],**  **p.[(Asp1165Gly)]; [(Gln3318*)]** | **chr7:21641082A>G,**  **chr7:21828886C>T** |
| --- | --- | --- |

Rare, predicted damaging missense **variant of uncertain significance** and likely pathogenic (PVS1, PM2) stopgain variant (MAF=0.0002 / 0), inherited *in trans*. *DNAH11* was associated with laterality defects and congenital heart defects [^94^](#_ENREF_94).

Family 087:

Proband with dextrocardia, left atrial isomerism, interrupted inferior vena cava (IVC) with azygous continuation of the IVC to right SVC, single right SVC drains to right-sided atrium, hepatic veins drain to right-sided atrium, all four pulmonary veins drain to right-sided atrium, unbalanced AVSD (large primum ASD, large VSD) with common atrioventricular valve committed to a large anterior left-sided morphological right ventricle, hypoplastic posterior and right-sided left ventricle, DORV with side-by-side great vessels (aorta leftward), left aortic arch. Non-cardiac features include left-sided liver, polysplenia, superior mesenteric vein to the right of the superior mesenteric artery, stomach to the right, abnormal configuration of the colon (likely malrotation), and macrocephaly. A clinical follow-up also showed mild respiratory issues and ultrastructural ciliry abnormalities through electron microscopy on nasal scrapings.

| ***DNAH9* (NM_001372.3)** | **c.[4421A>G];[13244T>C],**  **p.[(Asp1474Gly)];[(Ile4415Thr)]** | **chr17:11593560A>G,**  **chr17:11872627T>C** |
| --- | --- | --- |

Rare, predicted damaging missense variants of uncertain significance (PMS2, PP3), inherited in trans (MAF= 0.0001 / 0.000057). *DNAH9* was associated with laterality defects, ciliary dyskinesia, and complex heart defects [^95^](#_ENREF_95)^,^ [^96^](#_ENREF_96). Variant 1 was located in the dynein heavy chain domain 2, variant 2 was located in the ATP-binding dynein motor region D5, both affected conserved positions. The variants were deemed **causative** for primary ciliary dyskinesia through clinical re-assessment.

Family 089:

Proband with congenitally corrected TGA (ccTGA), moderate outlet VSD, pulmonary stenosis (valvar and supravalvar), left aortic arch, small secundum ASD, normal tricuspid valve, clonus, and clinodactyly.

| ***UBXN10* (NM_152376.3)** | **c.477_481delTGAAG, p.(Ser159Argfs*44)** | **chr1:20517528delAAGTG (homozygous)** |
| --- | --- | --- |

Homozygous, rare frameshift variant in a **candidate gene of uncertain significance** (MAF=0.000049). The encoded protein localized to cilia and was required for ciliogenesis. It interacted with the Intraflagellar Transport B (IFT-B) complex, which controlled anterograde traffic into cilia. Inhibition of UBXN10 in zebrafish embryos (morpholinos) caused defects in left-right symmetry. 20% of embryos had a heart tube positioned at the midline or on the right side. This phenotype could be partially rescued by injection of human *UBXN10* mRNA [^97^](#_ENREF_97).

Family 093:

Proband with dextrocardia, right atrial isomerism, complete balanced AVSD (common atrium, large inlet VSD), right ventricle is to left of left ventricle, right ventricle is the outlet ventricle with the remote aorta located anterior and leftward to the pulmonary artery, left-hand topology, bilateral SVC (right SVC to right-sided atrium, left SVC to left-sided atrium, no bridging vein), all four pulmonary veins to right-sided atrium unobstructed, IVC to right-sided atrium, pulmonary atresia, non-confluent pulmonary arteries supplied by bilateral ducts, right aortic arch, single coronary artery from right sinus. Extracardiac features include short stature, failure to thrive, borderline microcephaly, right-sided liver extends across midline, asplenia, stomach to the right, IVC and hepatic veins arise to the left of the aorta, right isomeric bronchi, and bilateral right atrial appendages.

| ***DNAH8* (NM_001206927.1)** | **c.[991A>G];[10773C>G],**  **p.[(Thr331Ala)];[(Phe3591Leu)]** | **chr6:38705623A>G,**  **chr6:38890944C>G** |
| --- | --- | --- |

Rare missense **variants of uncertain significance**, inherited *in trans* (MAF= 0.0003 / 0.0004). Variant 1 was predicted to potentially affect splicing, other prediction tools suggested the variant to be tolerated. Variant 2 was *in silico* predicted to be damaging.

| **3p11.2-3p12.3** | **Deletion (8.3 Mb)** | **chr3:79166228-87437174del** |
| --- | --- | --- |

Maternally inherited 8.3 Mb-deletion of uncertain significance, including genes: *CADM2*, *CHMP2B*, *GBE1*, *POU1F1*, *ROBO1*, *VGLL3*. *ROBO1* is a non-constrained (pLI=0) gene of uncertain significance, associated with incompletely penetrant septal defects and tetralogy of Fallot [^98^](#_ENREF_98). ROBO1 functions in the Slit/Robo pathway, which is involved in many aspects of heart development. Expression of Notch- and downstream genes was down-regulated in Robo1 mutants [^99^](#_ENREF_99).

Family 099:

Proband with tricuspid valve dysplasia and hypoplasia, hypoplastic right ventricle, large perimembranous VSD, large secundum ASD, ADHD, learning disability/mild intellectual disability, and periauricular skin tag.

| **3p26.1-3pter** | **Deletion (4.3 Mb), duplication (1.8 Mb)** | **chr3:60001-4354200del,**  **chr3:4360001-6187000dup** |
| --- | --- | --- |

Maternally inherited **deletion/duplication of uncertain significance**. Deleted genes were *CHL1*, *CNTN4*, *CNTN6*, *CRBN*, *IL5RA*, *LRRN1*, *SETMAR*, *TRNT1*. Duplicated genes were *ARL8B*, *BHLHE40*, *EDEM1*, *ITPR1*, *SUMF1*. The copy number variant overlapped a known contiguous gene deletion syndrome, characterized by global developmental delay, heart defects, and preauricular skin tags. *LRRN1* is a constrained gene (pLI=0.97), expressed in the secondary heart field and a candidate for outflow tract development [^100^](#_ENREF_100).

Family 101:

Proband with aortic coarctation, BAV, mildly hypoplastic aortic valve, failure to thrive, sparse hair, and multicystic left kidney.

| **20q13.2** | **Duplication (494 kb)** | **chr20:50307001-50801000dup** |
| --- | --- | --- |

Paternally inherited **duplication of uncertain inheritance**, including genes *ATP9A*, *SALL4*, *ZFP64*. *SALL4* haploinsufficiency was associated with limb, renal, and heart malformations, but it was also a candidate for cardiac malformations in larger 20q13.2 duplications [^101^](#_ENREF_101).

Family 103:

Proband with mild left ventricular hypoplasia, complete AVSD (large primum ASD, large inlet VSD) with common atrioventricualr valve (deficient left mural leaflet), bilateral SVC with no bridging vein (left SVC to coronary sinus), mild intrauterine growth retardation, dysmorphic features, lumbar scoliosis with a semi-segmented hemivertebra (L4) and an additional (6th) lumbar vertebrae. The father was diagnosed with bicuspid aortic valve at 34 years.

| ***SMARCC1* (NM_003074.3)** | **c.1844_1845delAAinsTAAG,**  **p.(Lys615Ilefs*49)** | **chr3:47712202delTTinsCTTA** |
| --- | --- | --- |

Paternally inherited frameshift variant in a **candidate gene of uncertain significance**. The highly constrained gene (pLI=1) encodes for a core subunit of the SWI/SNF complex [^102^](#_ENREF_102). In mouse, the gene was involved in embryonic development and growth [^103^](#_ENREF_103)^,^ [^104^](#_ENREF_104), including the regulation of angiogenesis [^105^](#_ENREF_105). Knockdown of a *SMARCC1* homologue in zebrafish was associated with various congenital defects, with heart defects being the most penetrant feature [^106^](#_ENREF_106). A *de novo* loss-of-function variant was identified in a patient with multiple congenital anomalies, including meningomyelocele, scoliosis, solitary kidney, imperforated anus, short neck, narrow chest, vertebral anomalies, and lower limb anomalies [^107^](#_ENREF_107). Defects in other SWI/SNF subunits were associated with developmental disorders, including heart defects: *ARID1A*, *ARID1B*, *ARID2*, *SMARCA4*, *SMARCB1*, *SMARCC2*, *SMARCE1*, *ACTL6A*, and *DPF2* [^108^](#_ENREF_108)^,^ [^109^](#_ENREF_109).

Family 148:

Proband with critical aortic stenosis, aortic coarctation, hypoplastic transverse arch, left aortic arch, large perimembranous VSD, partial anomalous pulmonary venous drainage (left upper pulmonary vein drains by a vertical vein to the innominate vein), functionally bicuspid aortic valve, mild mitral stenosis, developmental delay (gross motor, speech and language), failure to thrive, tracheal tug on inspiration, high palate, triangular face, ptosis, hyperteloric, protruding forward facing ears, sacral dimple, eczema, and dysmorphisms.

| ***KMT2D* (NM_003482.3)** | **c.15673C>T, p.(Arg5225Cys)** | **chr12:49420076G>A** |
| --- | --- | --- |

*De novo* **likely pathogenic** (PS2, PM2) variant for Kabuki syndrome. It was not reported in controls and *in silico* predicted to be damaging. The same variant was reported *de novo* in a patient with Kabuki syndrome [^110^](#_ENREF_110).

Family 149:

Proband with aortic coarctation, BAV, and arterial thrombosis (likely a consequence of surgery). The mother a normal research echocardiography, the father reported a normal clinical echocardiography.

| **2p13.1-2p12** | **Deletion (600 kb)** | **chr2:74646148-75240197** |
| --- | --- | --- |

Paternally inherited **deletion of uncertain significance**, including genes: *RTKN*, *WDR54*, *CCDC142*, *LBX2*, *DOK1*, *HTRA2*, *INO80B*, *MOGS*, *WBP1*, *C2orf81*, *SEMA4F*, *M1AP*, *LOXL3*, *TTC31*, *TLX2*, *DQX1*, *POLE4*, *MRPL53*, *PCGF1*, *HK2*, *AUP1*. *PCGF1* is constrained (pLI=1) and encodes a transcriptional repressor involved in ectoderm / mesoderm differentiation, and anterior-posterior patterning in early embryonic development. It also interacted with *BCOR* (associated with oculofaciocardiodental syndrome) [^111^](#_ENREF_111)^,^ [^112^](#_ENREF_112).

Family 157:

Proband with HLHS (mitral atresia, aortic atresia), global developmental delay, hypotonia, borderline short stature and gastroesophageal reflux.

| ***POGZ* (NM_015100.3)** | **c.3403delG, p.(Glu1135Argfs*3)** | **chr1:151378108delC** |
| --- | --- | --- |

*De novo* **pathogenic** (PVS1, PS2, PM2) variant. The gene was associated with White-Sutton syndrome, and *de novo* variants were identified in cohorts with congenital heart defects and neurodevelopmental disorders [^113-115^](#_ENREF_113).

Family 160:

Proband with TGA, aortic coarctation with transverse arch hypoplasia, hypoplastic non-apex forming right ventricle, small apical muscular VSD, coronary pattern: 1LCx2R.

| ***PTPN11* (NM_002834.4)** | **c.661A>G, p.(Ile221Val)** | **chr12:112893772A>G** |
| --- | --- | --- |

Paternally inherited, rare **variant of uncertain significance** (MAF= 0.000004). The variant was reported in a patient with Noonan syndrome, but neither segregation testing nor functional validation were done [^116^](#_ENREF_116). The missense substitution affected a highly conserved residue in the protein-tyrosine phosphatase-like domain. The proband was not found to have typical features of Noonan syndrome.

Family 168:

Proband with HLHS (mitral stenosis, aortic atresia) and short stature.

| ***TOP2A* (NM_001067.3)** | **c.2800-1G>A** | **chr17:38556662C>T** |
| --- | --- | --- |

Maternally inherited variant in a **candidate gene of uncertain significance**. The gene is constrained (pLI=0.99) and encodes a DNA topisomerase. The variant was absent in controls (MAF=0) and predicted to result in in-frame exon-skipping (67 amino acids: aa934-1000, including a DNA-binding domain [^117^](#_ENREF_117)). A *de novo* deletion of uncertain significance was reported in an individual with TOF [^118^](#_ENREF_118).


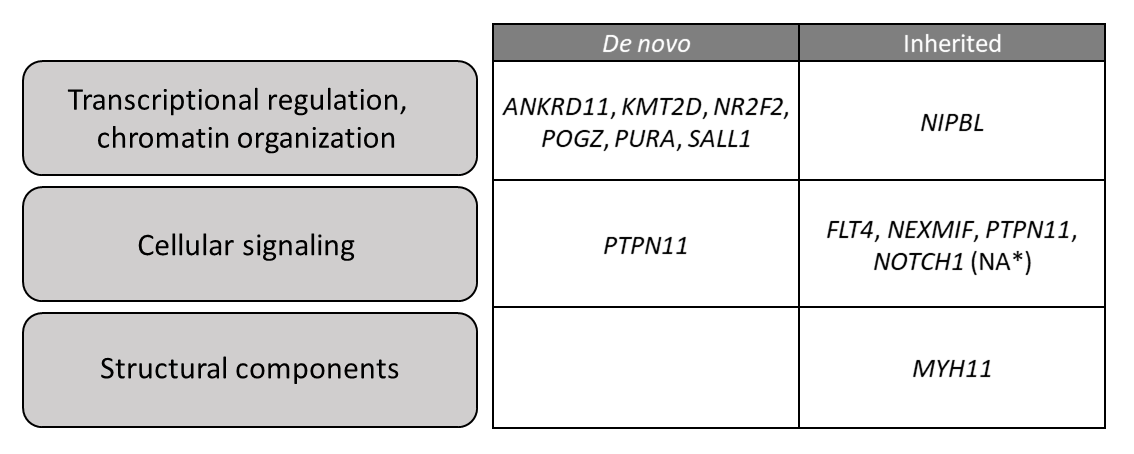


**Figure S2. Gene functions and inheritance.** Disease-associated genes were categorized according their biological function, based on literature review. Variants in this cohort were categorized as *de novo* or inherited.

* Inheritance of the NOTCH1 deletion was not assessed (NA), but the family history indicated that it was inherited from the affected father.

**Table S3. Comparison of selected genome-wide sequencing studies of cohorts with congenital heart disease.**

| **Study** | **Sequencing** | **Cohort size** | **Cohort characteristics** | **Diagnostic yield*** | **Diagnostic characteristics** |
| --- | --- | --- | --- | --- | --- |
| Reuter, Chaturvedi et al. | Genome | 111 (104 trios) | Pediatric, complex cardiac lesions, prior genetic testing | 13/111 (11.7%) | Genome-wide analysis, clinical-grade interpretation |
| Alankarage et al. (2018) | Genome | 97 (all trios) | Heterogeneous CHD requiring surgical correction | 30/97 (31%) | Genome-wide analysis (2 tiers) |
| Hauser et al. (2018) | Genome | 34 (all trios) | Neonatal and pediatric, heterogeneous CHD | 2/34 (6%) | Genome-wide analysis, clinical-grade interpretation |
| Lord et al. (2019) | Exome | 81 (mostly trios) | Fetuses with cardiac anomalies | 9/81 (11.1%) | 1,628 genes associated with developmental disorders, clinical-grade interpretation |
| Petrovski et al. (2019) | Exome | 77 (all trios) | Fetuses with cardiac anomalies | 4/77 (5.2%) | Genome-wide analysis, clinical-grade interpretation |
| Westphal et al. (2019) | Exome | 30 (27 trios) | Prenatally diagnosed CHD | 6/30 (20%) | Genome-wide analysis (3 tiers) |
| Szot et al. (2018) | Exome | 30 (all trios) | Heterogeneous CHD | 13/30 (43.3%) | Genome-wide analysis (2 tiers) |
| Jin et al. (2017) | Exome | 2,871 (2,645 trios) | Pediatric, heterogeneous CHD | NA; 10.1% with a putative genetic contribution | Genome-wide analysis |

* As indicated by the authors.

CHD, congenital heart disease; NA, not applicable.

**References**

1. Girdea M, Dumitriu S, Fiume M, Bowdin S, Boycott KM, Chenier S, Chitayat D, Faghfoury H, Meyn MS, Ray PN, So J, Stavropoulos DJ and Brudno M. PhenoTips: patient phenotyping software for clinical and research use. *Hum Mutat*. 2013;34:1057-65.

2. Li H and Durbin R. Fast and accurate short read alignment with Burrows-Wheeler transform. *Bioinformatics*. 2009;25:1754-60.

3. DePristo MA, Banks E, Poplin R, Garimella KV, Maguire JR, Hartl C, Philippakis AA, del Angel G, Rivas MA, Hanna M, McKenna A, Fennell TJ, Kernytsky AM, Sivachenko AY, Cibulskis K, Gabriel SB, Altshuler D and Daly MJ. A framework for variation discovery and genotyping using next-generation DNA sequencing data. *Nat Genet*. 2011;43:491-8.

4. Van der Auwera GA, Carneiro MO, Hartl C, Poplin R, Del Angel G, Levy-Moonshine A, Jordan T, Shakir K, Roazen D, Thibault J, Banks E, Garimella KV, Altshuler D, Gabriel S and DePristo MA. From FastQ data to high confidence variant calls: the Genome Analysis Toolkit best practices pipeline. *Curr Protoc Bioinformatics*. 2013;43:11 10 1-33.

5. Zhu M, Need AC, Han Y, Ge D, Maia JM, Zhu Q, Heinzen EL, Cirulli ET, Pelak K, He M, Ruzzo EK, Gumbs C, Singh A, Feng S, Shianna KV and Goldstein DB. Using ERDS to infer copy-number variants in high-coverage genomes. *Am J Hum Genet*. 2012;91:408-21.

6. Abyzov A, Urban AE, Snyder M and Gerstein M. CNVnator: an approach to discover, genotype, and characterize typical and atypical CNVs from family and population genome sequencing. *Genome Res*. 2011;21:974-84.

7. Trost B, Walker S, Wang Z, Thiruvahindrapuram B, MacDonald JR, Sung WWL, Pereira SL, Whitney J, Chan AJS, Pellecchia G, Reuter MS, Lok S, Yuen RKC, Marshall CR, Merico D and Scherer SW. A Comprehensive Workflow for Read Depth-Based Identification of Copy-Number Variation from Whole-Genome Sequence Data. *Am J Hum Genet*. 2018;102:142-155.

8. Chen X, Schulz-Trieglaff O, Shaw R, Barnes B, Schlesinger F, Kallberg M, Cox AJ, Kruglyak S and Saunders CT. Manta: rapid detection of structural variants and indels for germline and cancer sequencing applications. *Bioinformatics*. 2016;32:1220-2.

9. Wang K, Li M and Hakonarson H. ANNOVAR: functional annotation of genetic variants from high-throughput sequencing data. *Nucleic Acids Res*. 2010;38:e164.

10. Lek M, Karczewski KJ, Minikel EV, Samocha KE, Banks E, Fennell T, O'Donnell-Luria AH, Ware JS, Hill AJ, Cummings BB, Tukiainen T, Birnbaum DP, Kosmicki JA, Duncan LE, Estrada K, Zhao F, Zou J, Pierce-Hoffman E, Berghout J, Cooper DN, Deflaux N, DePristo M, Do R, Flannick J, Fromer M, Gauthier L, Goldstein J, Gupta N, Howrigan D, Kiezun A, Kurki MI, Moonshine AL, Natarajan P, Orozco L, Peloso GM, Poplin R, Rivas MA, Ruano-Rubio V, Rose SA, Ruderfer DM, Shakir K, Stenson PD, Stevens C, Thomas BP, Tiao G, Tusie-Luna MT, Weisburd B, Won HH, Yu D, Altshuler DM, Ardissino D, Boehnke M, Danesh J, Donnelly S, Elosua R, Florez JC, Gabriel SB, Getz G, Glatt SJ, Hultman CM, Kathiresan S, Laakso M, McCarroll S, McCarthy MI, McGovern D, McPherson R, Neale BM, Palotie A, Purcell SM, Saleheen D, Scharf JM, Sklar P, Sullivan PF, Tuomilehto J, Tsuang MT, Watkins HC, Wilson JG, Daly MJ, MacArthur DG and Exome Aggregation C. Analysis of protein-coding genetic variation in 60,706 humans. *Nature*. 2016;536:285-91.

11. Karczewski KJ, Francioli LC, Tiao G, Cummings BB, Alföldi J, Wang Q, Collins RL, Laricchia KM, Ganna A, Birnbaum DP, Gauthier LD, Brand H, Solomonson M, Watts NA, Rhodes D, Singer-Berk M, Seaby EG, Kosmicki JA, Walters RK, Tashman K, Farjoun Y, Banks E, Poterba T, Wang A, Seed C, Whiffin N, Chong JX, Samocha KE, Pierce-Hoffman E, Zappala Z, O'Donnell-Luria AH, Vallabh Minikel E, Weisburd B, Lek M, Ware JS, Vittal C, Armean IM, Bergelson L, Cibulskis K, Connolly KM, Covarrubias M, Donnelly S, Ferriera S, Gabriel S, Gentry J, Gupta N, Jeandet T, Kaplan D, Llanwarne C, Munshi R, Novod S, Petrillo N, Roazen D, Ruano-Rubio V, Saltzman A, Schleicher M, Soto J, Tibbetts K, Tolonen C, Wade G, Talkowski ME, Neale BM, Daly MJ and MacArthur DG. Variation across 141,456 human exomes and genomes reveals the spectrum of loss-of-function intolerance across human protein-coding genes. *bioRxiv*. 2019:531210.

12. Yuen RKC, Merico D, Bookman M, J LH, Thiruvahindrapuram B, Patel RV, Whitney J, Deflaux N, Bingham J, Wang Z, Pellecchia G, Buchanan JA, Walker S, Marshall CR, Uddin M, Zarrei M, Deneault E, D'Abate L, Chan AJ, Koyanagi S, Paton T, Pereira SL, Hoang N, Engchuan W, Higginbotham EJ, Ho K, Lamoureux S, Li W, MacDonald JR, Nalpathamkalam T, Sung WW, Tsoi FJ, Wei J, Xu L, Tasse AM, Kirby E, Van Etten W, Twigger S, Roberts W, Drmic I, Jilderda S, Modi BM, Kellam B, Szego M, Cytrynbaum C, Weksberg R, Zwaigenbaum L, Woodbury-Smith M, Brian J, Senman L, Iaboni A, Doyle-Thomas K, Thompson A, Chrysler C, Leef J, Savion-Lemieux T, Smith IM, Liu X, Nicolson R, Seifer V, Fedele A, Cook EH, Dager S, Estes A, Gallagher L, Malow BA, Parr JR, Spence SJ, Vorstman J, Frey BJ, Robinson JT, Strug LJ, Fernandez BA, Elsabbagh M, Carter MT, Hallmayer J, Knoppers BM, Anagnostou E, Szatmari P, Ring RH, Glazer D, Pletcher MT and Scherer SW. Whole genome sequencing resource identifies 18 new candidate genes for autism spectrum disorder. *Nat Neurosci*. 2017;20:602-611.

13. MacDonald JR, Ziman R, Yuen RK, Feuk L and Scherer SW. The Database of Genomic Variants: a curated collection of structural variation in the human genome. *Nucleic Acids Res*. 2014;42:D986-92.

14. Reuter MS, Jobling R, Chaturvedi RR, Manshaei R, Costain G, Heung T, Curtis M, Hosseini SM, Liston E, Lowther C, Oechslin E, Sticht H, Thiruvahindrapuram B, Mil SV, Wald RM, Walker S, Marshall CR, Silversides CK, Scherer SW, Kim RH and Bassett AS. Haploinsufficiency of vascular endothelial growth factor related signaling genes is associated with tetralogy of Fallot. *Genet Med*. 2019;21:1001-1007.

15. Ferrara N, Gerber HP and LeCouter J. The biology of VEGF and its receptors. *Nat Med*. 2003;9:669-76.

16. McTigue MA, Wickersham JA, Pinko C, Showalter RE, Parast CV, Tempczyk-Russell A, Gehring MR, Mroczkowski B, Kan CC, Villafranca JE and Appelt K. Crystal structure of the kinase domain of human vascular endothelial growth factor receptor 2: a key enzyme in angiogenesis. *Structure*. 1999;7:319-30.

17. Wilkinson S, Paterson HF and Marshall CJ. Cdc42-MRCK and Rho-ROCK signalling cooperate in myosin phosphorylation and cell invasion. *Nat Cell Biol*. 2005;7:255-61.

18. He PY, Yip WK, Chai BL, Chai BY, Jabar MF, Dusa N, Mohtarrudin N and Seow HF. Inhibition of cell migration and invasion by miR29a3p in a colorectal cancer cell line through suppression of CDC42BPA mRNA expression. *Oncol Rep*. 2017;38:3554-3566.

19. Martinelli S, Krumbach OHF, Pantaleoni F, Coppola S, Amin E, Pannone L, Nouri K, Farina L, Dvorsky R, Lepri F, Buchholzer M, Konopatzki R, Walsh L, Payne K, Pierpont ME, Vergano SS, Langley KG, Larsen D, Farwell KD, Tang S, Mroske C, Gallotta I, Di Schiavi E, Della Monica M, Lugli L, Rossi C, Seri M, Cocchi G, Henderson L, Baskin B, Alders M, Mendoza-Londono R, Dupuis L, Nickerson DA, Chong JX, University of Washington Center for Mendelian G, Meeks N, Brown K, Causey T, Cho MT, Demuth S, Digilio MC, Gelb BD, Bamshad MJ, Zenker M, Ahmadian MR, Hennekam RC, Tartaglia M and Mirzaa GM. Functional Dysregulation of CDC42 Causes Diverse Developmental Phenotypes. *Am J Hum Genet*. 2018;102:309-320.

20. Basagiannis D, Zografou S, Murphy C, Fotsis T, Morbidelli L, Ziche M, Bleck C, Mercer J and Christoforidis S. VEGF induces signalling and angiogenesis by directing VEGFR2 internalisation through macropinocytosis. *J Cell Sci*. 2016;129:4091-4104.

21. Ader F, De Groote P, Reant P, Rooryck-Thambo C, Dupin-Deguine D, Rambaud C, Khraiche D, Perret C, Pruny JF, Mathieu-Dramard M, Gerard M, Troadec Y, Gouya L, Jeunemaitre X, Van Maldergem L, Hagege A, Villard E, Charron P and Richard P. FLNC pathogenic variants in patients with cardiomyopathies: Prevalence and genotype-phenotype correlations. *Clin Genet*. 2019.

22. Roldan-Sevilla A, Palomino-Doza J, de Juan J, Sanchez V, Dominguez-Gonzalez C, Salguero-Bodes R and Arribas-Ynsaurriaga F. Missense Mutations in the FLNC Gene Causing Familial Restrictive Cardiomyopathy. *Circ Genom Precis Med*. 2019;12:e002388.

23. Valdes-Mas R, Gutierrez-Fernandez A, Gomez J, Coto E, Astudillo A, Puente DA, Reguero JR, Alvarez V, Moris C, Leon D, Martin M, Puente XS and Lopez-Otin C. Mutations in filamin C cause a new form of familial hypertrophic cardiomyopathy. *Nat Commun*. 2014;5:5326.

24. Tanaka AJ, Bai R, Cho MT, Anyane-Yeboa K, Ahimaz P, Wilson AL, Kendall F, Hay B, Moss T, Nardini M, Bauer M, Retterer K, Juusola J and Chung WK. De novo mutations in PURA are associated with hypotonia and developmental delay. *Cold Spring Harb Mol Case Stud*. 2015;1:a000356.

25. Lalani SR, Zhang J, Schaaf CP, Brown CW, Magoulas P, Tsai AC, El-Gharbawy A, Wierenga KJ, Bartholomew D, Fong CT, Barbaro-Dieber T, Kukolich MK, Burrage LC, Austin E, Keller K, Pastore M, Fernandez F, Lotze T, Wilfong A, Purcarin G, Zhu W, Craigen WJ, McGuire M, Jain M, Cooney E, Azamian M, Bainbridge MN, Muzny DM, Boerwinkle E, Person RE, Niu Z, Eng CM, Lupski JR, Gibbs RA, Beaudet AL, Yang Y, Wang MC and Xia F. Mutations in PURA cause profound neonatal hypotonia, seizures, and encephalopathy in 5q31.3 microdeletion syndrome. *Am J Hum Genet*. 2014;95:579-83.

26. Bubien V, Bonnet F, Brouste V, Hoppe S, Barouk-Simonet E, David A, Edery P, Bottani A, Layet V, Caron O, Gilbert-Dussardier B, Delnatte C, Dugast C, Fricker JP, Bonneau D, Sevenet N, Longy M, Caux F and French Cowden Disease N. High cumulative risks of cancer in patients with PTEN hamartoma tumour syndrome. *J Med Genet*. 2013;50:255-63.

27. Frazier TW, Embacher R, Tilot AK, Koenig K, Mester J and Eng C. Molecular and phenotypic abnormalities in individuals with germline heterozygous PTEN mutations and autism. *Mol Psychiatry*. 2015;20:1132-8.

28. Busch RM, Chapin JS, Mester J, Ferguson L, Haut JS, Frazier TW and Eng C. Cognitive characteristics of PTEN hamartoma tumor syndromes. *Genet Med*. 2013;15:548-53.

29. Heindl M, Handel N, Ngeow J, Kionke J, Wittekind C, Kamprad M, Rensing-Ehl A, Ehl S, Reifenberger J, Loddenkemper C, Maul J, Hoffmeister A, Aretz S, Kiess W, Eng C and Uhlig HH. Autoimmunity, intestinal lymphoid hyperplasia, and defects in mucosal B-cell homeostasis in patients with PTEN hamartoma tumor syndrome. *Gastroenterology*. 2012;142:1093-1096 e6.

30. Heald B, Mester J, Rybicki L, Orloff MS, Burke CA and Eng C. Frequent gastrointestinal polyps and colorectal adenocarcinomas in a prospective series of PTEN mutation carriers. *Gastroenterology*. 2010;139:1927-33.

31. Chen HH, Handel N, Ngeow J, Muller J, Huhn M, Yang HT, Heindl M, Berbers RM, Hegazy AN, Kionke J, Yehia L, Sack U, Blaser F, Rensing-Ehl A, Reifenberger J, Keith J, Travis S, Merkenschlager A, Kiess W, Wittekind C, Walker L, Ehl S, Aretz S, Dustin ML, Eng C, Powrie F and Uhlig HH. Immune dysregulation in patients with PTEN hamartoma tumor syndrome: Analysis of FOXP3 regulatory T cells. *J Allergy Clin Immunol*. 2017;139:607-620 e15.

32. Reijnders MRF, Leventer RJ, Lee BH, Baralle D, Selber P, Paciorkowski AR and Hunt D. PURA-Related Neurodevelopmental Disorders. In: M. P. Adam, H. H. Ardinger, R. A. Pagon, S. E. Wallace, L. J. H. Bean, K. Stephens and A. Amemiya, eds. *GeneReviews((R))* Seattle (WA); 1993.

33. Xu S, Fan Y, Sun Y, Wang L, Gu X and Yu Y. Targeted/exome sequencing identified mutations in ten Chinese patients diagnosed with Noonan syndrome and related disorders. *BMC Med Genomics*. 2017;10:62.

34. Page DJ, Miossec MJ, Williams SG, Monaghan RM, Fotiou E, Cordell HJ, Sutcliffe L, Topf A, Bourgey M, Bourque G, Eveleigh R, Dunwoodie SL, Winlaw DS, Bhattacharya S, Breckpot J, Devriendt K, Gewillig M, Brook JD, Setchfield KJ, Bu'Lock FA, O'Sullivan J, Stuart G, Bezzina CR, Mulder BJM, Postma AV, Bentham JR, Baron M, Bhaskar SS, Black GC, Newman WG, Hentges KE, Lathrop GM, Santibanez-Koref M and Keavney BD. Whole Exome Sequencing Reveals the Major Genetic Contributors to Nonsyndromic Tetralogy of Fallot. *Circ Res*. 2019;124:553-563.

35. Demion M, Thireau J, Gueffier M, Finan A, Khoueiry Z, Cassan C, Serafini N, Aimond F, Granier M, Pasquie JL, Launay P and Richard S. Trpm4 gene invalidation leads to cardiac hypertrophy and electrophysiological alterations. *PLoS One*. 2014;9:e115256.

36. Gueffier M, Zintz J, Lambert K, Finan A, Aimond F, Chakouri N, Hedon C, Granier M, Launay P, Thireau J, Richard S and Demion M. The TRPM4 channel is functionally important for the beneficial cardiac remodeling induced by endurance training. *J Muscle Res Cell Motil*. 2017;38:3-16.

37. Guinamard R, Demion M, Magaud C, Potreau D and Bois P. Functional expression of the TRPM4 cationic current in ventricular cardiomyocytes from spontaneously hypertensive rats. *Hypertension*. 2006;48:587-94.

38. Guinamard R and Bois P. Involvement of transient receptor potential proteins in cardiac hypertrophy. *Biochim Biophys Acta*. 2007;1772:885-94.

39. Earley S. TRPM4 channels in smooth muscle function. *Pflugers Arch*. 2013;465:1223-31.

40. Earley S, Straub SV and Brayden JE. Protein kinase C regulates vascular myogenic tone through activation of TRPM4. *Am J Physiol Heart Circ Physiol*. 2007;292:H2613-22.

41. Hill MA and Davis MJ. Coupling a change in intraluminal pressure to vascular smooth muscle depolarization: still stretching for an explanation. *Am J Physiol Heart Circ Physiol*. 2007;292:H2570-2.

42. Farhan MA, Azad AK, Touret N and Murray AG. FGD5 regulates VEGF receptor-2 coupling to PI3 kinase and receptor recycling. *Arterioscler Thromb Vasc Biol*. 2017;37:2301-2310.

43. Heldin J, O'Callaghan P, Hernandez Vera R, Fuchs PF, Gerwins P and Kreuger J. FGD5 sustains vascular endothelial growth factor A (VEGFA) signaling through inhibition of proteasome-mediated VEGF receptor 2 degradation. *Cell Signal*. 2017;40:125-132.

44. Lorenzo M, Stolte-Dijkstra I, van Rheenen P, Smith RG, Scheers T and Walia JS. Clinical spectrum of KIAA2022 pathogenic variants in males: Case report of two boys with KIAA2022 pathogenic variants and review of the literature. *Am J Med Genet A*. 2018;176:1455-1462.

45. de Lange IM, Helbig KL, Weckhuysen S, Moller RS, Velinov M, Dolzhanskaya N, Marsh E, Helbig I, Devinsky O, Tang S, Mefford HC, Myers CT, van Paesschen W, Striano P, van Gassen K, van Kempen M, de Kovel CG, Piard J, Minassian BA, Nezarati MM, Pessoa A, Jacquette A, Maher B, Balestrini S, Sisodiya S, Warde MT, De St Martin A, Chelly J, Euro E-RESMAEwg, van 't Slot R, Van Maldergem L, Brilstra EH and Koeleman BP. De novo mutations of KIAA2022 in females cause intellectual disability and intractable epilepsy. *J Med Genet*. 2016;53:850-858.

46. Sartoretto JL, Jin BY, Bauer M, Gertler FB, Liao R and Michel T. Regulation of VASP phosphorylation in cardiac myocytes: differential regulation by cyclic nucleotides and modulation of protein expression in diabetic and hypertrophic heart. *Am J Physiol Heart Circ Physiol*. 2009;297:H1697-710.

47. Ram R, Wescott AP, Varandas K, Dirksen RT and Blaxall BC. Mena associates with Rac1 and modulates connexin 43 remodeling in cardiomyocytes. *Am J Physiol Heart Circ Physiol*. 2014;306:H154-9.

48. Brancaccio M, Hirsch E, Notte A, Selvetella G, Lembo G and Tarone G. Integrin signalling: the tug-of-war in heart hypertrophy. *Cardiovasc Res*. 2006;70:422-33.

49. Liu Y, Dillon AR, Tillson M, Makarewich C, Nguyen V, Dell'Italia L, Sabri AK, Rizzo V and Tsai EJ. Volume overload induces differential spatiotemporal regulation of myocardial soluble guanylyl cyclase in eccentric hypertrophy and heart failure. *J Mol Cell Cardiol*. 2013;60:72-83.

50. Tanaka K, Ito M, Kodama M, Hoyano M, Kimura S, Mitsuma W, Hirono S, Adachi T, Watanabe K, Nakazawa M and Aizawa Y. Long-term carperitide treatment attenuates left ventricular remodeling in rats with heart failure after autoimmune myocarditis. *J Cardiovasc Pharmacol*. 2009;54:232-9.

51. Fiedler LR, Bachetti T, Leiper J, Zachary I, Chen L, Renne T and Wojciak-Stothard B. The ADMA/DDAH pathway regulates VEGF-mediated angiogenesis. *Arterioscler Thromb Vasc Biol*. 2009;29:2117-24.

52. Yuan B, Pehlivan D, Karaca E, Patel N, Charng WL, Gambin T, Gonzaga-Jauregui C, Sutton VR, Yesil G, Bozdogan ST, Tos T, Koparir A, Koparir E, Beck CR, Gu S, Aslan H, Yuregir OO, Al Rubeaan K, Alnaqeb D, Alshammari MJ, Bayram Y, Atik MM, Aydin H, Geckinli BB, Seven M, Ulucan H, Fenercioglu E, Ozen M, Jhangiani S, Muzny DM, Boerwinkle E, Tuysuz B, Alkuraya FS, Gibbs RA and Lupski JR. Global transcriptional disturbances underlie Cornelia de Lange syndrome and related phenotypes. *J Clin Invest*. 2015;125:636-51.

53. Jin SC, Homsy J, Zaidi S, Lu Q, Morton S, DePalma SR, Zeng X, Qi H, Chang W, Sierant MC, Hung WC, Haider S, Zhang J, Knight J, Bjornson RD, Castaldi C, Tikhonoa IR, Bilguvar K, Mane SM, Sanders SJ, Mital S, Russell MW, Gaynor JW, Deanfield J, Giardini A, Porter GA, Jr., Srivastava D, Lo CW, Shen Y, Watkins WS, Yandell M, Yost HJ, Tristani-Firouzi M, Newburger JW, Roberts AE, Kim R, Zhao H, Kaltman JR, Goldmuntz E, Chung WK, Seidman JG, Gelb BD, Seidman CE, Lifton RP and Brueckner M. Contribution of rare inherited and de novo variants in 2,871 congenital heart disease probands. *Nat Genet*. 2017;49:1593-1601.

54. Al Turki S, Manickaraj AK, Mercer CL, Gerety SS, Hitz MP, Lindsay S, D'Alessandro LC, Swaminathan GJ, Bentham J, Arndt AK, Louw J, Breckpot J, Gewillig M, Thienpont B, Abdul-Khaliq H, Harnack C, Hoff K, Kramer HH, Schubert S, Siebert R, Toka O, Cosgrove C, Watkins H, Lucassen AM, O'Kelly IM, Salmon AP, Bu'lock FA, Granados-Riveron J, Setchfield K, Thornborough C, Brook JD, Mulder B, Klaassen S, Bhattacharya S, Devriendt K, Fitzpatrick DF, Consortium UK, Wilson DI, Mital S and Hurles ME. Rare variants in NR2F2 cause congenital heart defects in humans. *Am J Hum Genet*. 2014;94:574-85.

55. Upadia J, Gonzales PR and Robin NH. Novel de novo pathogenic variant in the NR2F2 gene in a boy with congenital heart defect and dysmorphic features. *Am J Med Genet A*. 2018;176:1423-1426.

56. Bashamboo A, Eozenou C, Jorgensen A, Bignon-Topalovic J, Siffroi JP, Hyon C, Tar A, Nagy P, Solyom J, Halasz Z, Paye-Jaouen A, Lambert S, Rodriguez-Buritica D, Bertalan R, Martinerie L, Rajpert-De Meyts E, Achermann JC and McElreavey K. Loss of Function of the Nuclear Receptor NR2F2, Encoding COUP-TF2, Causes Testis Development and Cardiac Defects in 46,XX Children. *Am J Hum Genet*. 2018;102:487-493.

57. Goldenberg A, Riccardi F, Tessier A, Pfundt R, Busa T, Cacciagli P, Capri Y, Coutton C, Delahaye-Duriez A, Frebourg T, Gatinois V, Guerrot AM, Genevieve D, Lecoquierre F, Jacquette A, Khau Van Kien P, Leheup B, Marlin S, Verloes A, Michaud V, Nadeau G, Mignot C, Parent P, Rossi M, Toutain A, Schaefer E, Thauvin-Robinet C, Van Maldergem L, Thevenon J, Satre V, Perrin L, Vincent-Delorme C, Sorlin A, Missirian C, Villard L, Mancini J, Saugier-Veber P and Philip N. Clinical and molecular findings in 39 patients with KBG syndrome caused by deletion or mutation of ANKRD11. *Am J Med Genet A*. 2016;170:2847-2859.

58. Pace NP, Maggouta F, Twigden M and Borg I. Molecular cytogenetic characterisation of a novel de novo ring chromosome 6 involving a terminal 6p deletion and terminal 6q duplication in the different arms of the same chromosome. *Mol Cytogenet*. 2017;10:9.

59. Aldinger KA, Lehmann OJ, Hudgins L, Chizhikov VV, Bassuk AG, Ades LC, Krantz ID, Dobyns WB and Millen KJ. FOXC1 is required for normal cerebellar development and is a major contributor to chromosome 6p25.3 Dandy-Walker malformation. *Nat Genet*. 2009;41:1037-42.

60. Smith PL, Myers JT, Rogers CE, Zhou L, Petryniak B, Becker DJ, Homeister JW and Lowe JB. Conditional control of selectin ligand expression and global fucosylation events in mice with a targeted mutation at the FX locus. *J Cell Biol*. 2002;158:801-15.

61. Song Y, Willer JR, Scherer PC, Panzer JA, Kugath A, Skordalakes E, Gregg RG, Willer GB and Balice-Gordon RJ. Neural and synaptic defects in slytherin, a zebrafish model for human congenital disorders of glycosylation. *PLoS One*. 2010;5:e13743.

62. Nagy R, Ganapathi S, Comeras I, Peterson C, Orloff M, Porter K, Eng C, Ringel MD and Kloos RT. Frequency of germline PTEN mutations in differentiated thyroid cancer. *Thyroid*. 2011;21:505-10.

63. Sarquis MS, Agrawal S, Shen L, Pilarski R, Zhou XP and Eng C. Distinct expression profiles for PTEN transcript and its splice variants in Cowden syndrome and Bannayan-Riley-Ruvalcaba syndrome. *Am J Hum Genet*. 2006;79:23-30.

64. Pilarski R, Stephens JA, Noss R, Fisher JL and Prior TW. Predicting PTEN mutations: an evaluation of Cowden syndrome and Bannayan-Riley-Ruvalcaba syndrome clinical features. *J Med Genet*. 2011;48:505-12.

65. Gil A, Rodriguez-Escudero I, Stumpf M, Molina M, Cid VJ and Pulido R. A functional dissection of PTEN N-terminus: implications in PTEN subcellular targeting and tumor suppressor activity. *PLoS One*. 2015;10:e0119287.

66. Denning G, Jean-Joseph B, Prince C, Durden DL and Vogt PK. A short N-terminal sequence of PTEN controls cytoplasmic localization and is required for suppression of cell growth. *Oncogene*. 2007;26:3930-40.

67. Andres-Pons A, Rodriguez-Escudero I, Gil A, Blanco A, Vega A, Molina M, Pulido R and Cid VJ. In vivo functional analysis of the counterbalance of hyperactive phosphatidylinositol 3-kinase p110 catalytic oncoproteins by the tumor suppressor PTEN. *Cancer Res*. 2007;67:9731-9.

68. Furnari FB, Huang HJ and Cavenee WK. The phosphoinositol phosphatase activity of PTEN mediates a serum-sensitive G1 growth arrest in glioma cells. *Cancer Res*. 1998;58:5002-8.

69. Pilarski R. PTEN Hamartoma Tumor Syndrome: A Clinical Overview. *Cancers (Basel)*. 2019;11.

70. Hamada K, Sasaki T, Koni PA, Natsui M, Kishimoto H, Sasaki J, Yajima N, Horie Y, Hasegawa G, Naito M, Miyazaki J, Suda T, Itoh H, Nakao K, Mak TW, Nakano T and Suzuki A. The PTEN/PI3K pathway governs normal vascular development and tumor angiogenesis. *Genes Dev*. 2005;19:2054-65.

71. Renard M, Callewaert B, Baetens M, Campens L, MacDermot K, Fryns JP, Bonduelle M, Dietz HC, Gaspar IM, Cavaco D, Stattin EL, Schrander-Stumpel C, Coucke P, Loeys B, De Paepe A and De Backer J. Novel MYH11 and ACTA2 mutations reveal a role for enhanced TGFbeta signaling in FTAAD. *Int J Cardiol*. 2013;165:314-21.

72. Yang H, Luo M, Fu Y, Cao Y, Yin K, Li W, Meng C, Ma Y, Zhang J, Fan Y, Shu C, Chang Q and Zhou Z. Genetic testing of 248 Chinese aortopathy patients using a panel assay. *Sci Rep*. 2016;6:33002.

73. Zhu L, Vranckx R, Khau Van Kien P, Lalande A, Boisset N, Mathieu F, Wegman M, Glancy L, Gasc JM, Brunotte F, Bruneval P, Wolf JE, Michel JB and Jeunemaitre X. Mutations in myosin heavy chain 11 cause a syndrome associating thoracic aortic aneurysm/aortic dissection and patent ductus arteriosus. *Nat Genet*. 2006;38:343-9.

74. LaHaye S, Corsmeier D, Basu M, Bowman JL, Fitzgerald-Butt S, Zender G, Bosse K, McBride KL, White P and Garg V. Utilization of Whole Exome Sequencing to Identify Causative Mutations in Familial Congenital Heart Disease. *Circ Cardiovasc Genet*. 2016;9:320-9.

75. Manso AM, Li R, Monkley SJ, Cruz NM, Ong S, Lao DH, Koshman YE, Gu Y, Peterson KL, Chen J, Abel ED, Samarel AM, Critchley DR and Ross RS. Talin1 has unique expression versus talin 2 in the heart and modifies the hypertrophic response to pressure overload. *J Biol Chem*. 2013;288:4252-64.

76. Roca-Cusachs P, Gauthier NC, Del Rio A and Sheetz MP. Clustering of alpha(5)beta(1) integrins determines adhesion strength whereas alpha(v)beta(3) and talin enable mechanotransduction. *Proc Natl Acad Sci U S A*. 2009;106:16245-50.

77. Conti FJ, Monkley SJ, Wood MR, Critchley DR and Muller U. Talin 1 and 2 are required for myoblast fusion, sarcomere assembly and the maintenance of myotendinous junctions. *Development*. 2009;136:3597-606.

78. Qi L, Kolodziej T, Rajfur Z and Huang C. Roles of Talin2 in Traction Force Generation, Tumor Metastasis and Cardiovascular Integrity. *Curr Protein Pept Sci*. 2018;19:1071-1078.

79. Pitt SJ, Lam AK, Rietdorf K, Galione A and Sitsapesan R. Reconstituted human TPC1 is a proton-permeable ion channel and is activated by NAADP or Ca2+. *Sci Signal*. 2014;7:ra46.

80. Pitt SJ, Reilly-O'Donnell B and Sitsapesan R. Exploring the biophysical evidence that mammalian two-pore channels are NAADP-activated calcium-permeable channels. *J Physiol*. 2016;594:4171-9.

81. Brailoiu E, Churamani D, Cai X, Schrlau MG, Brailoiu GC, Gao X, Hooper R, Boulware MJ, Dun NJ, Marchant JS and Patel S. Essential requirement for two-pore channel 1 in NAADP-mediated calcium signaling. *J Cell Biol*. 2009;186:201-9.

82. Garcia-Rua V, Feijoo-Bandin S, Garcia-Vence M, Aragon-Herrera A, Bravo SB, Rodriguez-Penas D, Mosquera-Leal A, Lear PV, Parrington J, Alonso J, Rosello-Lleti E, Portoles M, Rivera M, Gonzalez-Juanatey JR and Lago F. Metabolic alterations derived from absence of Two-Pore Channel 1 at cardiac level. *J Biosci*. 2016;41:643-658.

83. Garcia-Rua V, Feijoo-Bandin S, Rodriguez-Penas D, Mosquera-Leal A, Abu-Assi E, Beiras A, Maria Seoane L, Lear P, Parrington J, Portoles M, Rosello-Lleti E, Rivera M, Gualillo O, Parra V, Hill JA, Rothermel B, Gonzalez-Juanatey JR and Lago F. Endolysosomal two-pore channels regulate autophagy in cardiomyocytes. *J Physiol*. 2016;594:3061-77.

84. Patel S and Kilpatrick BS. Two-pore channels and disease. *Biochim Biophys Acta Mol Cell Res*. 2018;1865:1678-1686.

85. Garcia-Rua V, Otero MF, Lear PV, Rodriguez-Penas D, Feijoo-Bandin S, Noguera-Moreno T, Calaza M, Alvarez-Barredo M, Mosquera-Leal A, Parrington J, Brugada J, Portoles M, Rivera M, Gonzalez-Juanatey JR and Lago F. Increased expression of fatty-acid and calcium metabolism genes in failing human heart. *PLoS One*. 2012;7:e37505.

86. She J, Guo J, Chen Q, Zeng W, Jiang Y and Bai XC. Structural insights into the voltage and phospholipid activation of the mammalian TPC1 channel. *Nature*. 2018;556:130-134.

87. Sridhara SC, Carvalho S, Grosso AR, Gallego-Paez LM, Carmo-Fonseca M and de Almeida SF. Transcription Dynamics Prevent RNA-Mediated Genomic Instability through SRPK2-Dependent DDX23 Phosphorylation. *Cell Rep*. 2017;18:334-343.

88. Nowak DG, Amin EM, Rennel ES, Hoareau-Aveilla C, Gammons M, Damodoran G, Hagiwara M, Harper SJ, Woolard J, Ladomery MR and Bates DO. Regulation of vascular endothelial growth factor (VEGF) splicing from pro-angiogenic to anti-angiogenic isoforms: a novel therapeutic strategy for angiogenesis. *J Biol Chem*. 2010;285:5532-40.

89. Wang HY, Lin W, Dyck JA, Yeakley JM, Songyang Z, Cantley LC and Fu XD. SRPK2: a differentially expressed SR protein-specific kinase involved in mediating the interaction and localization of pre-mRNA splicing factors in mammalian cells. *J Cell Biol*. 1998;140:737-50.

90. Keilhack H, David FS, McGregor M, Cantley LC and Neel BG. Diverse biochemical properties of Shp2 mutants. Implications for disease phenotypes. *J Biol Chem*. 2005;280:30984-93.

91. Petrovski S, Aggarwal V, Giordano JL, Stosic M, Wou K, Bier L, Spiegel E, Brennan K, Stong N, Jobanputra V, Ren Z, Zhu X, Mebane C, Nahum O, Wang Q, Kamalakaran S, Malone C, Anyane-Yeboa K, Miller R, Levy B, Goldstein DB and Wapner RJ. Whole-exome sequencing in the evaluation of fetal structural anomalies: a prospective cohort study. *Lancet*. 2019;393:758-767.

92. Evans IM, Kennedy SA, Paliashvili K, Santra T, Yamaji M, Lovering RC, Britton G, Frankel P, Kolch W and Zachary IC. Vascular endothelial growth factor (VEGF) promotes assembly of the p130Cas interactome to drive endothelial chemotactic signaling and angiogenesis. *Mol Cell Proteomics*. 2017;16:168-180.

93. Yamaoka-Tojo M, Ushio-Fukai M, Hilenski L, Dikalov SI, Chen YE, Tojo T, Fukai T, Fujimoto M, Patrushev NA, Wang N, Kontos CD, Bloom GS and Alexander RW. IQGAP1, a novel vascular endothelial growth factor receptor binding protein, is involved in reactive oxygen species--dependent endothelial migration and proliferation. *Circ Res*. 2004;95:276-83.

94. Liu S, Chen W, Zhan Y, Li S, Ma X, Ma D, Sheng W and Huang G. DNAH11 variants and its association with congenital heart disease and heterotaxy syndrome. *Sci Rep*. 2019;9:6683.

95. Loges NT, Antony D, Maver A, Deardorff MA, Gulec EY, Gezdirici A, Nothe-Menchen T, Hoben IM, Jelten L, Frank D, Werner C, Tebbe J, Wu K, Goldmuntz E, Cuturilo G, Krock B, Ritter A, Hjeij R, Bakey Z, Pennekamp P, Dworniczak B, Brunner H, Peterlin B, Tanidir C, Olbrich H, Omran H and Schmidts M. Recessive DNAH9 Loss-of-Function Mutations Cause Laterality Defects and Subtle Respiratory Ciliary-Beating Defects. *Am J Hum Genet*. 2018;103:995-1008.

96. Fassad MR, Shoemark A, Legendre M, Hirst RA, Koll F, le Borgne P, Louis B, Daudvohra F, Patel MP, Thomas L, Dixon M, Burgoyne T, Hayes J, Nicholson AG, Cullup T, Jenkins L, Carr SB, Aurora P, Lemullois M, Aubusson-Fleury A, Papon JF, O'Callaghan C, Amselem S, Hogg C, Escudier E, Tassin AM and Mitchison HM. Mutations in Outer Dynein Arm Heavy Chain DNAH9 Cause Motile Cilia Defects and Situs Inversus. *Am J Hum Genet*. 2018;103:984-994.

97. Raman M, Sergeev M, Garnaas M, Lydeard JR, Huttlin EL, Goessling W, Shah JV and Harper JW. Systematic proteomics of the VCP-UBXD adaptor network identifies a role for UBXN10 in regulating ciliogenesis. *Nat Cell Biol*. 2015;17:1356-69.

98. Kruszka P, Tanpaiboon P, Neas K, Crosby K, Berger SI, Martinez AF, Addissie YA, Pongprot Y, Sittiwangkul R, Silvilairat S, Makonkawkeyoon K, Yu L, Wynn J, Bennett JT, Mefford HC, Reynolds WT, Liu X, Mommersteeg MTM, Chung WK, Lo CW and Muenke M. Loss of function in ROBO1 is associated with tetralogy of Fallot and septal defects. *J Med Genet*. 2017;54:825-829.

99. Mommersteeg MT, Yeh ML, Parnavelas JG and Andrews WD. Disrupted Slit-Robo signalling results in membranous ventricular septum defects and bicuspid aortic valves. *Cardiovasc Res*. 2015;106:55-66.

100. Barth JL, Clark CD, Fresco VM, Knoll EP, Lee B, Argraves WS and Lee KH. Jarid2 is among a set of genes differentially regulated by Nkx2.5 during outflow tract morphogenesis. *Dev Dyn*. 2010;239:2024-33.

101. Briand-Suleau A, Martinovic J, Tosca L, Tou B, Brisset S, Bouligand J, Delattre V, Giurgea I, Bachir J, Folliot P, Goumy C, Francannet C, Guiochon-Mantel A, Benachi A, Vermeesch J, Tachdjian G, Vago P, Goossens M and Metay C. SALL4 and NFATC2: two major actors of interstitial 20q13.2 duplication. *Eur J Med Genet*. 2014;57:174-80.

102. Wang W, Xue Y, Zhou S, Kuo A, Cairns BR and Crabtree GR. Diversity and specialization of mammalian SWI/SNF complexes. *Genes Dev*. 1996;10:2117-30.

103. Nguyen H, Sokpor G, Pham L, Rosenbusch J, Stoykova A, Staiger JF and Tuoc T. Epigenetic regulation by BAF (mSWI/SNF) chromatin remodeling complexes is indispensable for embryonic development. *Cell Cycle*. 2016;15:1317-24.

104. Tuoc TC, Narayanan R and Stoykova A. BAF chromatin remodeling complex: cortical size regulation and beyond. *Cell Cycle*. 2013;12:2953-9.

105. Han D, Jeon S, Sohn DH, Lee C, Ahn S, Kim WK, Chung H and Seong RH. SRG3, a core component of mouse SWI/SNF complex, is essential for extra-embryonic vascular development. *Dev Biol*. 2008;315:136-46.

106. Scott CA, Marsden AN, Rebagliati MR, Zhang Q, Chamling X, Searby CC, Baye LM, Sheffield VC and Slusarski DC. Nuclear/cytoplasmic transport defects in BBS6 underlie congenital heart disease through perturbation of a chromatin remodeling protein. *PLoS Genet*. 2017;13:e1006936.

107. Al Mutairi F, Alzahrani F, Ababneh F, Kashgari AA and Alkuraya FS. A mendelian form of neural tube defect caused by a de novo null variant in SMARCC1 in an identical twin. *Ann Neurol*. 2018;83:433-436.

108. Machol K, Rousseau J, Ehresmann S, Garcia T, Nguyen TTM, Spillmann RC, Sullivan JA, Shashi V, Jiang YH, Stong N, Fiala E, Willing M, Pfundt R, Kleefstra T, Cho MT, McLaughlin H, Rosello Piera M, Orellana C, Martinez F, Caro-Llopis A, Monfort S, Roscioli T, Nixon CY, Buckley MF, Turner A, Jones WD, van Hasselt PM, Hofstede FC, van Gassen KLI, Brooks AS, van Slegtenhorst MA, Lachlan K, Sebastian J, Madan-Khetarpal S, Sonal D, Sakkubai N, Thevenon J, Faivre L, Maurel A, Petrovski S, Krantz ID, Tarpinian JM, Rosenfeld JA, Lee BH, Undiagnosed Diseases N and Campeau PM. Expanding the Spectrum of BAF-Related Disorders: De Novo Variants in SMARCC2 Cause a Syndrome with Intellectual Disability and Developmental Delay. *Am J Hum Genet*. 2019;104:164-178.

109. Bevilacqua A, Willis MS and Bultman SJ. SWI/SNF chromatin-remodeling complexes in cardiovascular development and disease. *Cardiovasc Pathol*. 2014;23:85-91.

110. Bogershausen N, Gatinois V, Riehmer V, Kayserili H, Becker J, Thoenes M, Simsek-Kiper PO, Barat-Houari M, Elcioglu NH, Wieczorek D, Tinschert S, Sarrabay G, Strom TM, Fabre A, Baynam G, Sanchez E, Nurnberg G, Altunoglu U, Capri Y, Isidor B, Lacombe D, Corsini C, Cormier-Daire V, Sanlaville D, Giuliano F, Le Quan Sang KH, Kayirangwa H, Nurnberg P, Meitinger T, Boduroglu K, Zoll B, Lyonnet S, Tzschach A, Verloes A, Di Donato N, Touitou I, Netzer C, Li Y, Genevieve D, Yigit G and Wollnik B. Mutation Update for Kabuki Syndrome Genes KMT2D and KDM6A and Further Delineation of X-Linked Kabuki Syndrome Subtype 2. *Hum Mutat*. 2016;37:847-64.

111. Gearhart MD, Corcoran CM, Wamstad JA and Bardwell VJ. Polycomb group and SCF ubiquitin ligases are found in a novel BCOR complex that is recruited to BCL6 targets. *Mol Cell Biol*. 2006;26:6880-9.

112. Junco SE, Wang R, Gaipa JC, Taylor AB, Schirf V, Gearhart MD, Bardwell VJ, Demeler B, Hart PJ and Kim CA. Structure of the polycomb group protein PCGF1 in complex with BCOR reveals basis for binding selectivity of PCGF homologs. *Structure*. 2013;21:665-71.

113. Homsy J, Zaidi S, Shen Y, Ware JS, Samocha KE, Karczewski KJ, DePalma SR, McKean D, Wakimoto H, Gorham J, Jin SC, Deanfield J, Giardini A, Porter GA, Jr., Kim R, Bilguvar K, Lopez-Giraldez F, Tikhonova I, Mane S, Romano-Adesman A, Qi H, Vardarajan B, Ma L, Daly M, Roberts AE, Russell MW, Mital S, Newburger JW, Gaynor JW, Breitbart RE, Iossifov I, Ronemus M, Sanders SJ, Kaltman JR, Seidman JG, Brueckner M, Gelb BD, Goldmuntz E, Lifton RP, Seidman CE and Chung WK. De novo mutations in congenital heart disease with neurodevelopmental and other congenital anomalies. *Science*. 2015;350:1262-6.

114. White J, Beck CR, Harel T, Posey JE, Jhangiani SN, Tang S, Farwell KD, Powis Z, Mendelsohn NJ, Baker JA, Pollack L, Mason KJ, Wierenga KJ, Arrington DK, Hall M, Psychogios A, Fairbrother L, Walkiewicz M, Person RE, Niu Z, Zhang J, Rosenfeld JA, Muzny DM, Eng C, Beaudet AL, Lupski JR, Boerwinkle E, Gibbs RA, Yang Y, Xia F and Sutton VR. POGZ truncating alleles cause syndromic intellectual disability. *Genome Med*. 2016;8:3.

115. Stessman HAF, Willemsen MH, Fenckova M, Penn O, Hoischen A, Xiong B, Wang T, Hoekzema K, Vives L, Vogel I, Brunner HG, van der Burgt I, Ockeloen CW, Schuurs-Hoeijmakers JH, Klein Wassink-Ruiter JS, Stumpel C, Stevens SJC, Vles HS, Marcelis CM, van Bokhoven H, Cantagrel V, Colleaux L, Nicouleau M, Lyonnet S, Bernier RA, Gerdts J, Coe BP, Romano C, Alberti A, Grillo L, Scuderi C, Nordenskjold M, Kvarnung M, Guo H, Xia K, Piton A, Gerard B, Genevieve D, Delobel B, Lehalle D, Perrin L, Prieur F, Thevenon J, Gecz J, Shaw M, Pfundt R, Keren B, Jacquette A, Schenck A, Eichler EE and Kleefstra T. Disruption of POGZ Is Associated with Intellectual Disability and Autism Spectrum Disorders. *Am J Hum Genet*. 2016;98:541-552.

116. Lepri FR, Scavelli R, Digilio MC, Gnazzo M, Grotta S, Dentici ML, Pisaneschi E, Sirleto P, Capolino R, Baban A, Russo S, Franchin T, Angioni A and Dallapiccola B. Diagnosis of Noonan syndrome and related disorders using target next generation sequencing. *BMC Med Genet*. 2014;15:14.

117. Wendorff TJ, Schmidt BH, Heslop P, Austin CA and Berger JM. The structure of DNA-bound human topoisomerase II alpha: conformational mechanisms for coordinating inter-subunit interactions with DNA cleavage. *J Mol Biol*. 2012;424:109-24.

118. Glessner JT, Bick AG, Ito K, Homsy J, Rodriguez-Murillo L, Fromer M, Mazaika E, Vardarajan B, Italia M, Leipzig J, DePalma SR, Golhar R, Sanders SJ, Yamrom B, Ronemus M, Iossifov I, Willsey AJ, State MW, Kaltman JR, White PS, Shen Y, Warburton D, Brueckner M, Seidman C, Goldmuntz E, Gelb BD, Lifton R, Seidman J, Hakonarson H and Chung WK. Increased frequency of de novo copy number variants in congenital heart disease by integrative analysis of single nucleotide polymorphism array and exome sequence data. *Circ Res*. 2014;115:884-896.
